# First quantification of subtidal community structure at Tristan da Cunha Islands in the remote South Atlantic: from kelp forests to the deep sea

**DOI:** 10.1371/journal.pone.0195167

**Published:** 2018-03-29

**Authors:** Jennifer E. Caselle, Scott L. Hamilton, Kathryn Davis, Christopher D. H. Thompson, Alan Turchik, Ryan Jenkinson, Doug Simpson, Enric Sala

**Affiliations:** 1 Marine Science Institute, University of California, Santa Barbara, CA, United States of America; 2 Moss Landing Marine Laboratories, Moss Landing, CA, United States of America; 3 Centre for Marine Futures, University of Western Australia M092, Crawley, WA AUS; 4 Pristine Seas, National Geographic Society, Washington, DC, United States of America; 5 School of Journalism, University of Montana, Missoula, MT, United States of America; Sveriges lantbruksuniversitet, SWEDEN

## Abstract

Tristan da Cunha Islands, an archipelago of four rocky volcanic islands situated in the South Atlantic Ocean and part of the United Kingdom Overseas Territories (UKOTs), present a rare example of a relatively unimpacted temperate marine ecosystem. We conducted the first quantitative surveys of nearshore kelp forests, offshore pelagic waters and deep sea habitats. Kelp forests had very low biodiversity and species richness, but high biomass and abundance of those species present. Spatial variation in assemblage structure for both nearshore fish and invertebrates/algae was greatest between the three northern islands and the southern island of Gough, where sea temperatures were on average 3-4^o^ colder. Despite a lobster fishery that provides the bulk of the income to the Tristan islands, lobster abundance and biomass are comparable to or greater than many Marine Protected Areas in other parts of the world. Pelagic camera surveys documented a rich biodiversity offshore, including large numbers of juvenile blue sharks, *Prionace glauca*. Species richness and abundance in the deep sea is positively related to hard rocky substrate and biogenic habitats such as sea pens, crinoids, whip corals, and gorgonians were present at 40% of the deep camera deployments. We observed distinct differences in the deep fish community above and below ~750 m depth. Concurrent oceanographic sampling showed a discontinuity in temperature and salinity at this depth. While currently healthy, Tristan’s marine ecosystem is not without potential threats: shipping traffic leading to wrecks and species introductions, pressure to increase fishing effort beyond sustainable levels and the impacts of climate change all could potentially increase in the coming years. The United Kingdom has committed to protection of marine environments across the UKOTs, including Tristan da Cunha and these results can be used to inform future management decisions as well as provide a baseline against which future monitoring can be based.

## Introduction

In many places around the globe, the health of our oceans has declined over the past several decades due to a myriad of human-induced stressors, including overfishing, habitat destruction, pollution, invasive species, and climate change [1–6]. There are very few places left on the planet that are immune to anthropogenic impacts in some shape or form [7]. Remote locations provide one of the last opportunities to develop a baseline of the status and health of marine ecosystems where human impacts are minimized [8–10]. These quantitative baselines can provide unique opportunities to study future changes in population abundance, community structure, and ecosystem function through time, in addition to current spatial comparisons with locations that have experienced greater anthropogenic impacts.

Numerous pristine or near pristine systems have been studied in the tropics [8, 11–13] and in polar systems [14–17], highlighting the importance of bottom up forcing by oceanographic conditions and top down control by apex predators for structuring species assemblages. In contrast, very few subtropical to temperate systems exist in a near pristine state, with the exception of a number of remote islands in the Southern ocean (e.g., Prince Edward Islands). The likely reason for this is a long history of human habitation and exploitation in temperate regions, including the intensive harvesting and bycatch impacts on many fishes, pinnipeds, cetaceans, turtles, and sea birds, from even the most remote islands over the last two centuries [7, 18, 19].

One relatively pristine temperate marine system occurs in Tristan da Cunha Islands, an archipelago of 4 rocky volcanic islands situated in the South Atlantic Ocean, which is part of the United Kingdom Overseas Territories. The main island of Tristan da Cunha has been inhabited by upwards of 300 people since the late 1800s, while the islands of Inaccessible, Nightingale, and Gough have remained uninhabited throughout much of their history. While not free of human impacts, the marine ecosystem is thought to remain in a healthy state due to low levels of exploitation of most species. The Tristan economy is based on a fishery for the endemic Tristan rock lobster (*Jasus tristani*), which provides approximately 80% of the income for local islanders [20]; this fishery is MSC certified as sustainable. Additional species of reef fish are caught as for subsistence fishing and limited commercial fishing occurs at seamounts within the EEZ, however the fishing intensity is thought to be light on most stocks at present, particularly at the outer islands. Extreme weather conditions and a small exposed harbor presently limit access of local islanders to fishing offshore waters throughout most of the year. Additional threats in the marine environment involve invasive fish and invertebrate species, some of which became established with the grounding of a large oil rig in 2006 on Tristan and the wreck of the cargo ship MS Oliva on Nightingale in 2011 [21, 22].

The Tristan Islands are located roughly at the boundary between the Southern Ocean and the South Atlantic and sit at the confluence of two major ocean currents. The northern group (Tristan, Inaccessible and Nightingale) is located north of the Subtropical Convergence (STC) which is characterized by sharp differences in sea temperature and salinity [23] while Gough sits on or below this front depending on the season. Consequently, Gough Island sea surface temperature (SST) is on average 3–4°C colder than the northern islands and likely experiences enhanced nutrient availability. While the exact position and width of the STC varies [23, 24], these types of 'fronts' are locations of enhanced productivity [25, 26] and often are hotspots for marine organisms such as sharks, pinnipeds, cetaceans, and seabirds due to aggregations of prey species [27–34]. Fishery species also tend to aggregate at fronts [35, 36], increasing the probability of direct (bycatch, entanglement) and indirect (competition for prey) negative interactions between fisheries and marine megafauna and seabirds (reviewed in [37]).

A number of scientific expeditions have explored the nearshore and deeper waters (down to 1000 m) around the islands in the archipelago [38, 39]; however, comprehensive standardized quantitative surveys of marine assemblages from all islands have never been undertaken, particularly for multiple marine habitats. As part of a National Geographic Pristine Seas expedition, in partnership with the Tristan Government and the Royal Society for the Protection of Birds, we conducted an expedition to Tristan da Cunha in January—February of 2017. The goals for marine science research on the expedition were: (1) to conduct the first quantitative surveys in nearshore, pelagic, and deep sea habitats, (2) to assess spatial differences in the abundance and biomass of fishes, invertebrates, and kelps and associations among key players in the nearshore food web and (3) to provide information to aid in the spatial management of key resources and fisheries throughout the archipelago.

## Materials and methods

### Study region

Tristan da Cunha is a group of islands in the South Atlantic, representing the summits of massive shield volcanoes arising from abyssal depths. The archipelago lies over 2,700 km from South Africa and 3,700 km from the nearest shores of South America. The island of Saint Helena is the closest land, 2,400 km away to the north. This makes the Tristan archipelago one of the most geographically isolated island groups in the world. The main island, Tristan da Cunha (37°04’ S, 12°18’ W), is a circular volcanic cone that rises abruptly from the Atlantic Ocean (with eruptions recorded as recently as 2004). The island is 12 km in diameter (96 km^2^) and over 2,000 m at its summit. A narrow coastal plain in the northwest of the island supports a human population of ~270 people in a single settlement, ‘Edinburgh of the Seven Seas’, making it the world’s most remote inhabited island. Three main islands form the rest of the archipelago. Inaccessible (14 km^2^; 37°18’ S, 12°40’ W) and Nightingale (4 km^2^; 37°25’ S, 12°19’ W) Islands lie approximately 30 km to the southwest and south of Tristan da Cunha respectively. Gough Island (65 km^2^; 40°19’ S, 9°57’ W) is the most isolated in the group and lies 380 km to the southeast. Gough and Inaccessible Islands have been given World Heritage Site status for their near-pristine environments and vast populations of breeding seabirds and pinnipeds.

There have been several scientific expeditions to the Tristan da Cunha islands over the past century and a half, most conducting limited sampling or surveys [39]. The most notable efforts to exhaustively document marine biodiversity were the PhD thesis of Tim Andrew (1995) which described the fishes present in the waters of the archipelago, and a Darwin Initiative project that documented species diversity across multiple habitats and taxa [38, 39]. They describe the nearshore rocky reefs of the Tristan da Cunha group, which are characterized by underwater forests of the giant kelp, *Macrocystis pyrifera*, reaching depths up to 30–40 meters, and the understory pale kelp, *Laminaria pallida*. The diversity of marine life varies greatly with taxonomic group, but is generally low, given the remoteness of the archipelago. Fishes and many invertebrate groups, including crustaceans and echinoderms are species-poor, especially in nearshore habitats, while other groups, such as sponges and bryozoans, are more diverse. Seaweeds, in contrast, appear to be relatively diverse compared to other taxonomic groups. In deeper areas, very little is known about the distribution of habitat or species assemblages, and how they vary with depth, particularly below 200–300 m. For a complete review of the marine and terrestrial ecology, biodiversity, and conservation threats for the Tristan da Cunha archipelago, see Scott [39].

### Oceanography

A RBR concerto conductivity, temperature, and depth sensing unit (CTD) coupled with JFE Advantek O_2_ sensor was attached to the Deep Ocean drop-cams (see below) to measure vertical profiles of oceanographic characteristics at each island. A total of 11 CTD profiles were made and all islands were sampled (Gough *n* = 3; Inaccessible *n* = 1; Nightingale *n* = 2; Tristan da Cunha *n* = 5). Data were downloaded directly from the instrument and processed using the RBR software.

### Visual SCUBA survey techniques

**Fig 1:**
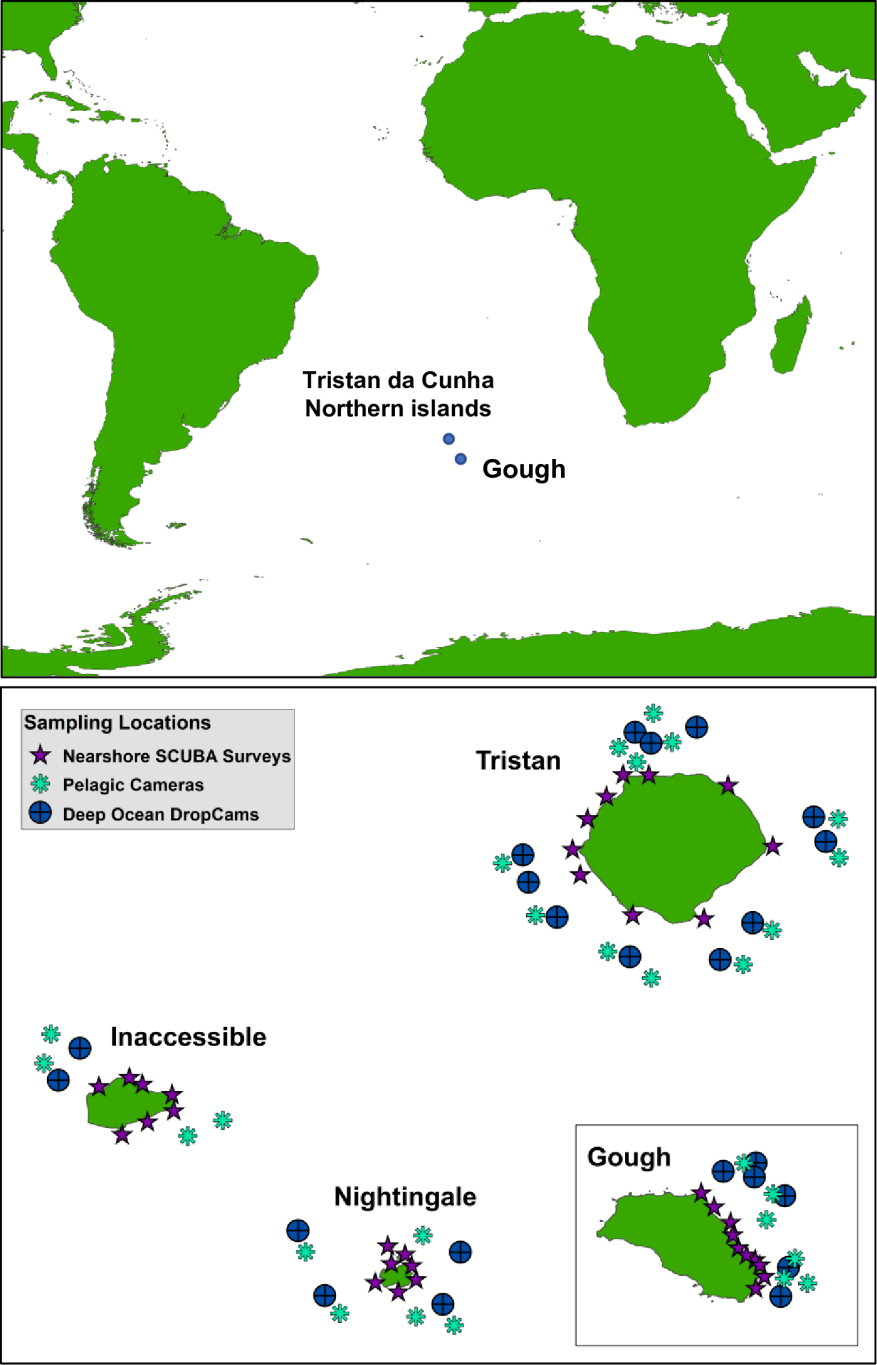
Map of the study region. Map depicts the locations of the Tristan da Cunha Islands in the South Atlantic (top) and locations of scuba surveys (*n* = 34), pelagic camera surveys (*n* = 26), and deep sea benthic camera surveys (*n* = 23) in the four islands of the Tristan da Cunha Islands group (bottom).

To characterize the nearshore marine environment, we employed visual SCUBA surveys based on a modification of sampling methods developed by the Partnership for Interdisciplinary Studies of Coastal Oceans (PISCO; www.piscoweb.org). PISCO survey methods have been widely used in rocky reef and kelp forest monitoring throughout the world [40], allowing for direct comparisons across locations. Sampling sites on each island were selected with the help of Landsat kelp canopy data to identify where kelp forests (and thus rocky habitat) have historically existed at each island as well as expert local help. Sites were allocated around each island in a stratified uniform design, with the spacing between sites dictated by island size (sites were spread further apart on bigger islands) the presence of appropriate rocky habitat, and expedition logistics (Fig 1). On Gough island, inclement wind and swell conditions limited the spatial survey effort to more sheltered shores.

At each survey site, divers quantified kelps (*Macrocystis* and *Laminaria*), macroinvertebrates, and fishes in two depth strata, positioned at 10 and 20 m depths. At each depth strata per site we ran 2 belt transects parallel to shore that were 30 m in length (*n* = 4 transects per site). While laying out the tape, one diver counted and estimated the sizes of all fishes to the nearest 1 cm along a 30 x 2 m transect. Counts along the transects extended to the surface or as far as visibility allowed, to include species associated with the kelp canopy and water column. At the end of each fish transect, the diver returned along the transect line, counting the number of giant kelp (*Macrocystis pyrifera*) plants and the number of stipes per plant, in addition to the number of pale kelp (*Laminaria pallida*) individuals within the 30 x 2 m transect area. Size thresholds were used to exclude juveniles of both species (*M*. *pyrifera* was counted if the stipe was >1 m in length, while *L*. *pallida* when the stipe length was >10 cm). Finally, the fish observer characterized the substrate type and vertical relief at each meter along the transect line using uniform point contact methodology (*n* = 30 points). The substrate was classified as bedrock, boulder (10cm-1m in the longest diameter), cobble (< 10cm), or sand, while vertical relief was classified as the largest vertical distance between the highest and lowest points in a 0.5 x 1 m box surrounding the point. Relief categories were defined as: 0–10 cm, 10–100 cm, 1-2m, and >2m. A second diver proceeded to count and estimate the size of all Tristan rock lobster *Jasus tristani* to the nearest 1 cm (carapace length) within a 30 x 8 m transect along the same line. At the end of the lobster transect, that diver swam back along the tape counting other conspicuous macroinvertebrates (sea urchins, sea stars, etc.) in a 30 x 2 m swath. In total, *n* = 34 sites were surveyed by SCUBA across the four islands (Fig 1).

### Pelagic and deep drop camera survey techniques

Mid-water baited remote underwater video systems (BRUVS) are designed to quantify pelagic fish assemblages [41]. These pelagic BRUVS consisted of a cross bar with two GoPro cameras fixed 0.8 m apart on an inward convergent angle of 8^o^. In longline formation, three units were deployed concurrently and separated by 200 m. Rigs were baited with 800 g of crushed fish and were deployed 2–4 km offshore of each island, with sampling sites stratified by island group and shore (Fig 1). Pelagic BRUVS were deployed for 2–4 hours at each sampling site. Using the software Event Measure, we identified all species to the lowest taxonomic level possible, and measured relative abundance (MaxN; maximum number of individuals observed in any given video frame [42]) and individual fork lengths. From this we generated species richness (the sum of individual species observed per sample) and total abundance (the sum of MaxN across all species observed per sample). Site means were calculated by taking the mean of the three camera units for that site. In total, *n* = 26 pelagic camera stations were sampled across the four islands.

National Geographic’s Exploration Technology Lab has developed Deep Ocean Drop-cams, which are high definition cameras (Sony Handycam HDR-XR520V 12 megapixel) encased in a borosilicate glass sphere that are rated to 10,000 m depth. Viewing area per frame is between 2–6 m^2^, depending on the steepness of the slope where the Drop-cam lands. Cameras were baited and deployed for 2–4 hours. Lighting at depth is achieved through a high intensity LED array directed using external reflectors. Depth gauging is accomplished using an external pressure sensor. The Drop-cams are weighted with a 22 kg external weight as an anchor with a descent rate of 1.5 m s^-1^. The primary release mechanism from the anchor is a burn wire that is activated using onboard battery voltage. The Drop-cams are positively buoyant resulting in an ascent rate of 0.5 m s^-1^. Drop-cams have an onboard VHF transmitter that allows for recovery using locating antennae with backup location achieved via communication with the ARGOS satellite system. The Deep Ocean drop-cams were deployed at sites on each side of every island (Fig 1), when conditions allowed and in a variety of depths on each island (S1 Table). In total, *n* = 23 deep-water camera stations were sampled across the 4 islands. For each drop, the camera rig was baited with 1 kg of frozen mackerel bait and the cameras and lights were turned on for 40 min, off for 30 min, and back on for another 30 min. Relative abundance of each species (MaxN) was measured as described above.

### Data analysis

To estimate fish and lobster biomass from kelp forest surveys, counts were tallied by length class and individual-specific lengths converted to body weights using the allometric length-weight conversion: W = a L^b^, where parameters a and b are species-specific constants, L is total length for fishes and carapace length for lobsters in cm, and W is weight in grams. Length-weight fitting parameters were obtained from FishBase [43] for fishes and Glass [20] for lobsters at the Tristan da Cunha islands, with the product of individual weights and numerical densities used to estimate biomass density by species. Numerical density (abundance) was expressed as number of individuals per 100 m^2^ and biomass was expressed as tonnes (t) per hectare (ha). We used linear mixed models to test for spatial differences in abundance and biomass of fishes, macroinvertebrates, and kelps, and percent cover of substrate characteristics at the island scale. These models compared variability among islands (fixed), sites (random), and depth zones (fixed), using sites nested within islands, and depth zones nested within sites and islands. We used a Restricted Maximum Likelihood Estimation (REML) method to obtain the residual variance estimate for the random factor (JMP Pro 13). All variables were square root transformed to improve normality and spread of residuals. To examine multivariate differences in the composition of fish and benthic assemblages among islands, we used the site-level mean densities for each species. Nonmetric multidimensional scaling (nMDS) analyses were used to visualize variation in fish and benthic communities among islands. Species abundances were square root transformed prior to analysis and a Bray-Curtis similarity matrix was used. PERMANOVA tests with the single factor of Island were used to statistically test for differences in fish and benthic communities among islands. We used SIMPER analyses to show the average percent dissimilarity in fish and benthic community structure (separate analyses using density) among pairs of islands and the percent contribution of particular species to community dissimilarity.

Mean total abundance and mean species richness of pelagic fish assemblages from midwater BRUVS were tested for differences among islands using univariate PERMANOVA analyses. We then tested the effect of island on assemblage structure using species abundances and biomasses. We also used PERMANOVA models and displayed the main drivers of patterns with canonical analysis of principal coordinates (CAP) plots. For these multivariate analyses, species abundances were square root transformed prior to calculation of Bray-Curtis similarity matrices.

To test for variation in diversity and community patterns in the deepwater habitats, the drop camera deployments were categorized into two different depth zones (0–750 m and >750 m depth) and two different benthic types (primarily soft bottom and primarily hard bottom). We tested the importance of these factors on species richness and total abundance with T-tests while multivariate assemblage patterns were visualized using nMDS and tested with PERMANOVA (using presence-absence data and the single factor of depth). All multivariate analyses described above were conducted in PRIMER v.6.

This field study was conducted with permission from the Director of Fisheries, Tristan da Cunha, Mr. James Glass after review of all protocols. No IACUC permission was required for this observational study.

## Results

### Temperature, salinity and dissolved oxygen

**Fig 2:**
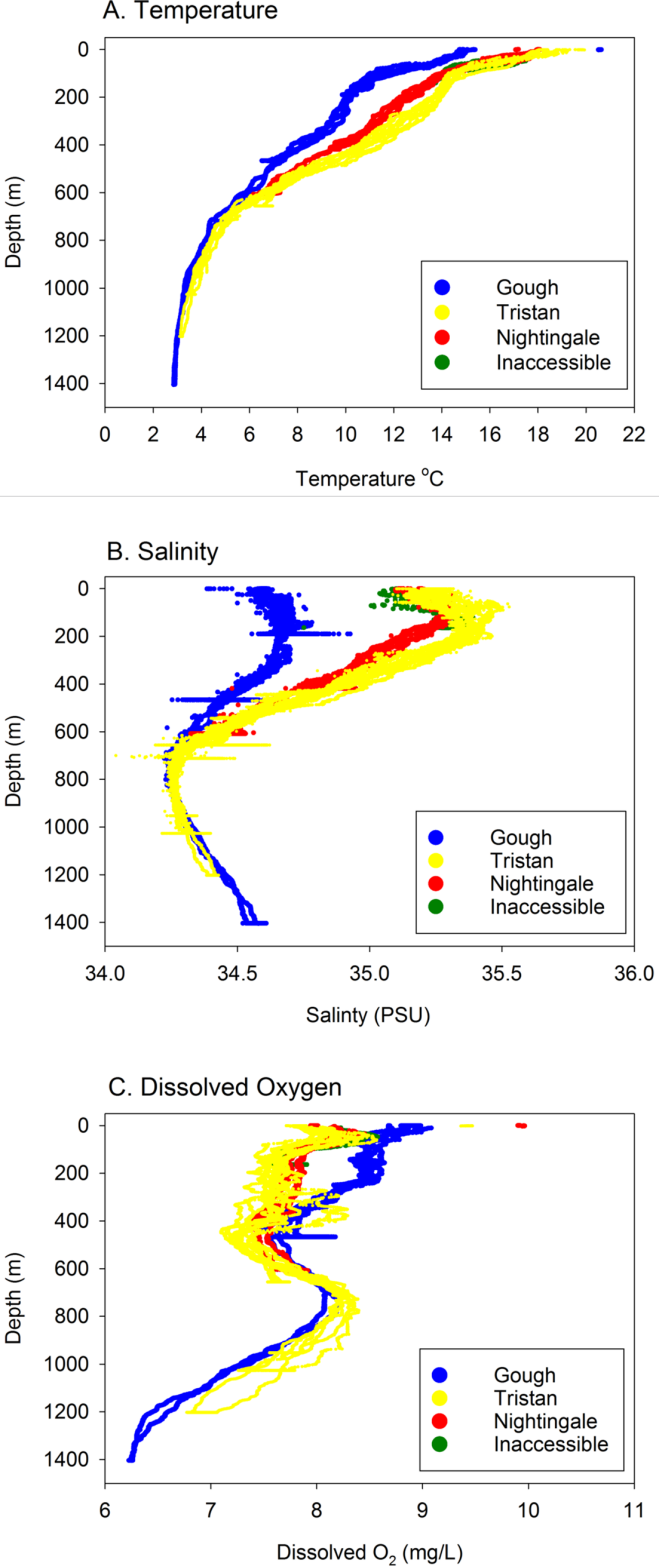
Temperature, salinity and dissolved oxygen. Oceanographic properties of the water column from a CTD attached to the deep-water drop cameras. A. Temperature (^o^C), B. salinity (PSU) and C. dissolved oxygen (mg/L) from each island by shore orientation are shown. The depth at which both temperature and salinity equilibrated among islands (approximately 600-750m) is shown in the grey band.

We measured properties of the water column with a CTD attached to the deep water drop cameras at all islands (Gough *n* = 3; Inaccessible *n* = 1; Nightingale *n* = 2; Tristan da Cunha *n* = 5). Sea temperature and salinity were different at Gough relative to the northern islands; Gough waters were colder and less saline (Fig 2A and 2B). Surface (3-10m) mean water temperatures were 14.9°C at Gough, 17.9°C at both Inaccessible and Nightingale, and 18.6°C at Tristan da Cunha. The depth at which both temperature and salinity equilibrated among islands was approximately 600–750 m, which matched the depth range where we observed a divergence in community structure from the deep-water drop cameras. Nightingale Island, which had similar surface temperature to Tristan da Cunha, diverged and became colder and less saline at approximately 100 m depth and remained so to about 500 m. Inaccessible may have shown the same pattern as Nightingale but our only drop at that island extended to 164 m. Patterns of dissolved oxygen were more difficult to interpret and less variable among islands (Fig 2C).

### Nearshore kelp forest surveys

#### Fish biomass and density

**Fig 3:**
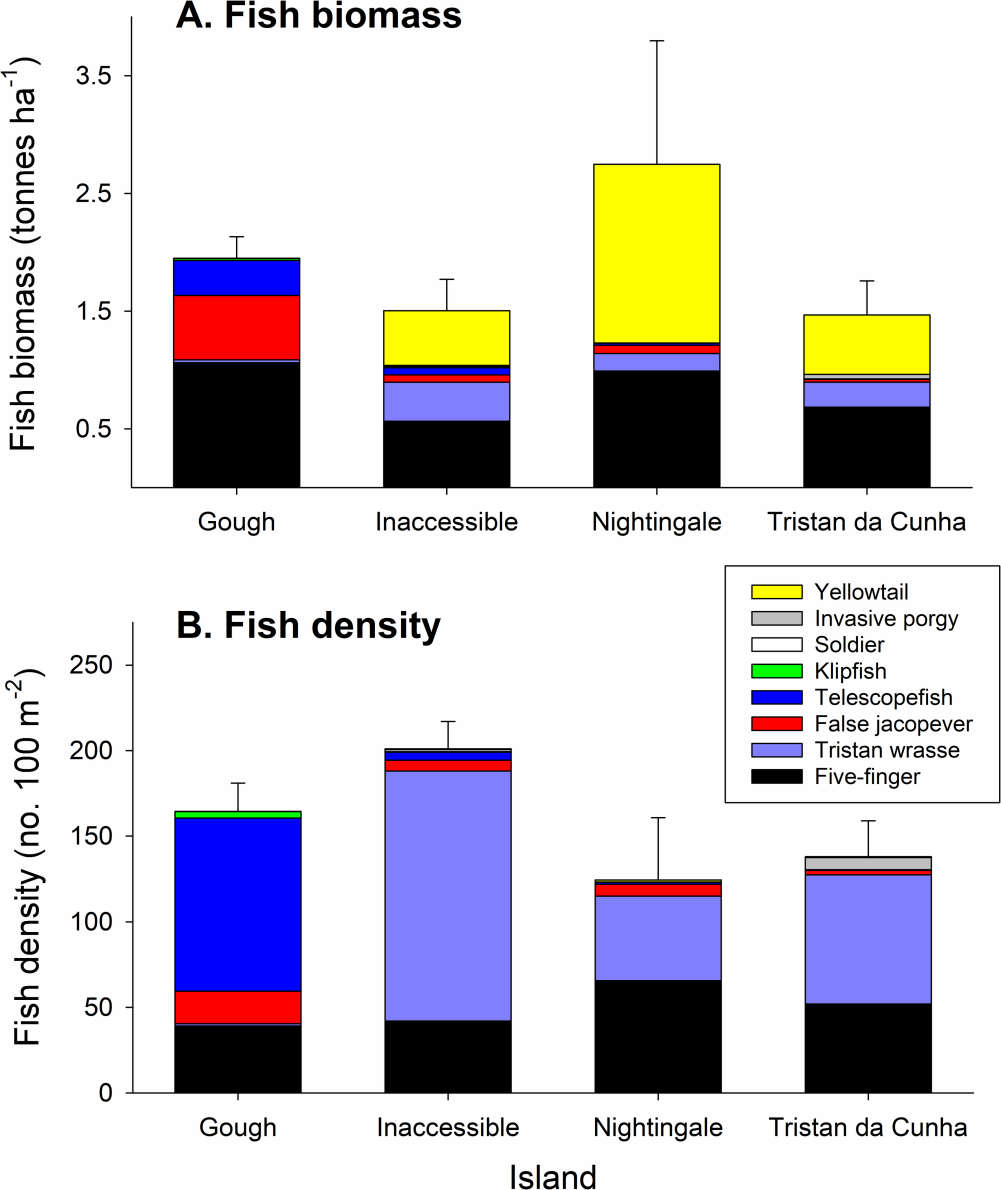
Fish biomass and density-island means. Stacked bar plots depicting fish biomass and density patterns in nearshore habitats of the Tristan da Cunha Islands from SCUBA surveys. Shown are the island-level mean (A) fish biomass (tonnes ha^-1^) and (B) fish density (no. m^-2^) for each species observed. Error bars are ± 1 standard error of the mean for the total fish biomass or total fish density respectively.

**Fig 4:**
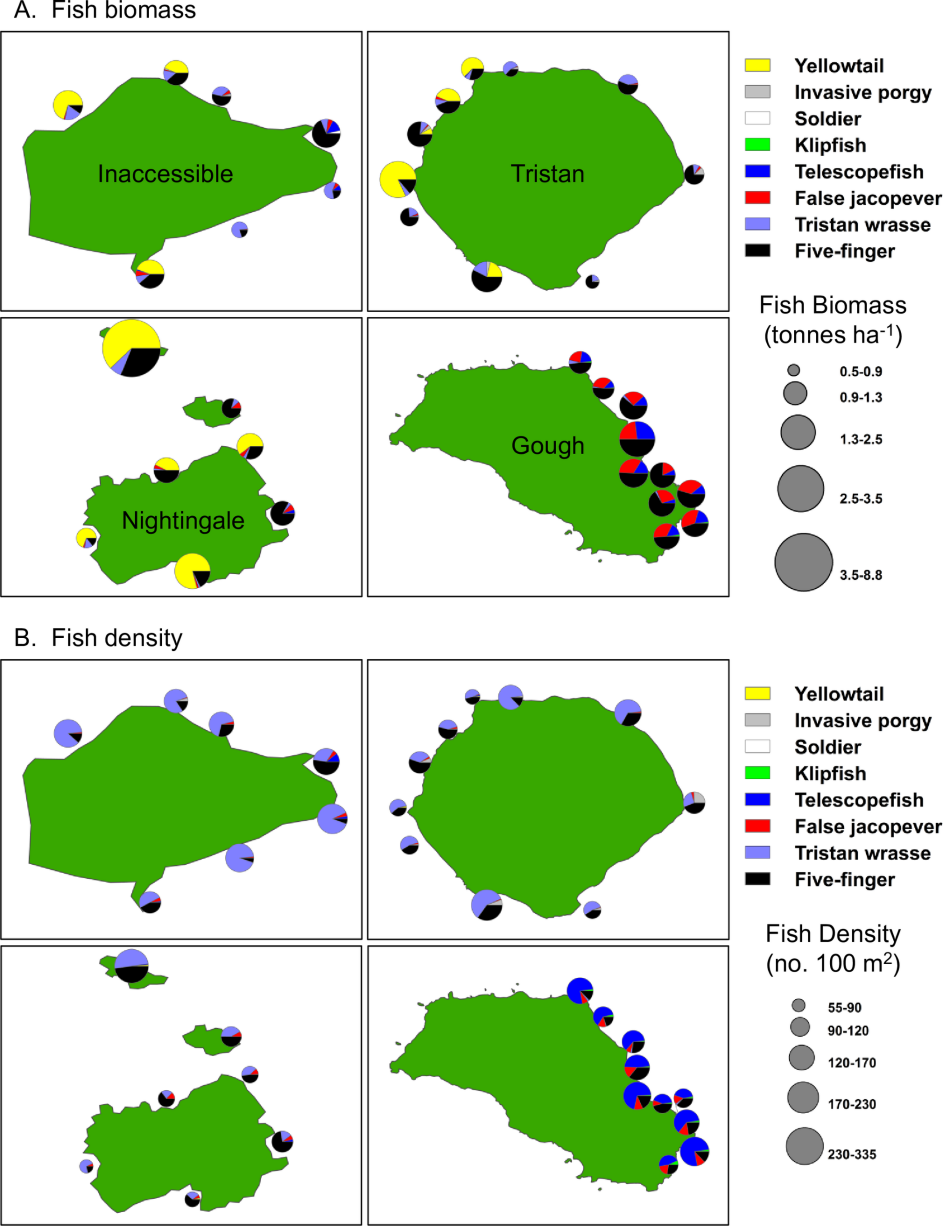
Fish biomass and density-site means. Bubble plots depicting site-level variation in fish biomass, density, and species composition of kelp forest fish communities in the four Tristan da Cunha Islands. Shown are plots of (A) fish biomass and (B) fish density. Bubble size scales with the biomass or density estimated using site-level means of SCUBA surveys.

In general, the Tristan da Cunha islands have very low biodiversity in nearshore marine habitats, with 8 species of fishes observed in kelp forests down to 20 m depth, but high biomass and abundance of those species (Fig 3, S2 Table). Total fish biomass (tonnes ha^-1^) was not significantly different among the four islands (Table 1, Fig 3A). Nightingale had the greatest fish biomass, largely driven by yellowtail (*Seriola lalandi*), which are highly mobile and transient in the kelp forest. Total fish biomass differed significantly among sites nested within islands (Table 1, Fig 4A). Inaccessible showed the least variation in total fish biomass among sites (ranging from 0.7 to 2.3 tonnes ha^-1^). Nightingale had the greatest site-to-site variation in total fish biomass (ranging from 1.0 to 8.8 tonnes ha^-1^) due to very high biomass (primarily of yellowtail) at a site located on Stoltenhoff Island, slightly offshore of the main island (Fig 4A). There was no effect of depth zone on total biomass (Table 2). Fish density (no. 100 m^-2^) differed significantly as a function of site (within island) and depth zone (nested within island and site) (Table 2, Fig 3B). The highest numerical abundance was recorded at Inaccessible, followed by Gough. Inaccessible showed the least variation among sites (ranging from 138.8 to 261.3 fish 100 m^-2^) while Nightingale showed the most variation in density among sites (54.2 to 333.3 fish 100 m^-2^) again, largely driven by a single site on Stoltenhoff Island (Fig 3B). Total fish density was greater on the deep zone transects (190.1 ± 14.3 fish 100 m^-2^) than the shallow zone transects (121.1 ± 11.3 fish 100 m^-2^).

**Table 1:** Linear mixed models testing the effects of island (fixed), site (random), and depth zone (fixed) on patterns of fish biomass among the Tristan da Cunha islands. Density and biomass were square root transformed. Statistically significant p-values are in bold text. Presented for the random effect of site are Wald p-values and percent of total variance explained.

	Model r^2^	Factors	df	F-ratio	P-value	Variance explained %
**A. Total fish**						
	0.76	Island	3	1.33	0.2820	
		Site[Island]			**0.0447**	22.8
		Zone[Site, Island]	34	0.97	0.5328	
**B. Five-finger (*Nemadactylus monodactylus*)**						
	0.83	Island	3	12.35	0.1636	
		Site[Island]		6.76	**0.001**	59.0
		Zone[Site, Island]	34	3.98	**<0.0001**	
**C. Tristan wrasse (*Suezichthys ornatus*)**						
	0.85	Island	3	58.35	**0.0004**	
		Site[Island]		7.10	**0.0009**	60.4
		Zone[Site, Island]	34	3.43	**<0.001**	
**D. False jacopever (*Sebastes capensis*)**						
	0.87	Island	3	140.00	**<0.001**	
		Site[Island]		2.25	**0.038**	23.9
		Zone[Site, Island]	34	1.08	0.38	
**E. Telescopefish (*Mendosoma lineatum*)**						
	0.81	Island	3	10.41	**<0.001**	
		Site[Island]		1.05	0.86	1.4
		Zone[Site, Island]	34	1.34	0.148	
**F. Klipfish (*Bovicthus diacanthus*)**			**3**			
	0.76	Island	3	54.53	**<0.0001**	
		Site[Island]		2.30	**0.0351**	24.6
		Zone[Site, Island]	34	1.30	0.1736	
**G. Invasive porgy (*Diplodus argenteus*)**						
	0.90	Island	3	23.86	**0.0120**	
		Site[Island]		5.52	**0.0016**	53,1
		Zone[Site, Island]	34	5.24	**<0.001**	
**H. Yellowtail (*Seriola lalandi*)**						
	0.39	Island	3	4.46	**0.0464**	
		Site[Island]		1.49	0.2442	10.9
		Zone[Site, Island]	34	0.73	0.8337	

**Table 2:** Linear mixed models testing the effects of island (fixed), site (random), and depth zone (fixed) on patterns of fish density among the Tristan da Cunha islands. Density and biomass were square root transformed. Statistically significant p-values are in bold text. Presented for the random effect of site are Wald p-values and percent of total variance explained.

	Model r^2^	Factors	df	F-ratio	P-value	Variance explained %
**A. Total fish**						
	0.79	Island	3	1.92	0.1464	
		Site[Island]			**0.0076**	37.1
		Zone[Site, Island]	34	3.99	**<0.001**	
**B. Five-finger (*Nemadactylus monodactylus*)**						
	0.83	Island	3	1.23	0.3139	
		Site[Island]			**0.0010**	59.2
		Zone[Site, Island]	34	3.71	**<0.001**	
**C. Tristan wrasse (*Suezichthys ornatus*)**						
	0.87	Island	3	13.20	**<0.001**	
		Site[Island]			**0.0020**	51.0
		Zone[Site, Island]	34	2.34	**0.0014**	
**D. False jacopever (*Sebastes capensis*)**						
	0.88	Island	3	30.68	**< .0001**	
		Site[Island]			**0.0061**	39.1
		Zone[Site, Island]	34	2.43	**0.0009**	
**E. Telescopefish (*Mendosoma lineatum*)**						
	0.84	Island	3	29.65	**<0.001**	
		Site[Island]			0.1693	13.5
		Zone[Site, Island]	34	5.92	**<0.001**	
**F. Klipfish (*Bovicthus diacanthus*)**						
	0.81	Island	3	37.07	**<0.001**	
		Site[Island]			0.1689	13.5
		Zone[Site, Island]	34	1.99	**0.0080**	
**G. Invasive porgy (*Diplodus argenteus*)**						
	0.90	Island	3	2.90	**0.0513**	
		Site[Island]			**0.0006**	67.8
		Zone[Site, Island]	34	8.01	**<0.0001**	
**H. Yellowtail (*Seriola lalandi*)**						
	0.53	Island	3	2.55	**0.0766**	
		Site[Island]			0.2948	9.6
		Zone[Site, Island]	34	0.74	0.8353	

There were significant island-to-island differences in the biomass and density of the majority of individual fish species tested (Tables 1 & 2). Most of this variation was driven by differences between the northern islands and Gough (Figs 3 & 4). Five-finger (*Nemadactylus monodactylus)* were both numerically abundant and composed a large part of the biomass at all four islands, but average sizes were larger in size at Gough (S1 Fig). Though not significantly different among islands, yellowtail (*Seriola lalandi*) were abundant at the northern islands but absent at Gough. At Gough, where most fish were larger on average (S1 Fig), false jacopever (*Sebastes capensis*) and telescope fish (*Mendosoma lineatum*) dominated the biomass estimates. The most numerically abundant fish species at the northern islands was the endemic Tristan wrasse (*Suezichthys ornatus*), with higher mean densities than the Five-finger. At Gough, telescope fish were the numerically dominant species, often found in large schools of juvenile-sized fishes. All species except for telescope fish, yellowtail and the less common klipfish (*Bovicthus diacanthus*) differed significantly in biomass and density (density only for klipfish) among sites within islands. Yellowtail are known to be highly mobile (at scales of these islands) and telescope fish mobility is unknown, but may play a role in the homogeneity across sites. Depth zone mattered less for biomass than for density. Depth zone explained significant variance in biomass of Five Finger, Tristan wrasse (higher biomass on deep transects) and the invasive Silver porgy (*Diplodus agenteus*) (higher biomass on shallow transects) but explained significant levels of variance in density for all species except Yellowtail (Tables 1 & 2). In general, telescope fish, Tristan wrasse, five finger, false jacopever, and yellowtail were all present in greater density in the deep zone, while klipfish and silver porgy were more abundant in the shallow zone.

#### Invertebrate and kelp biomass and density

**Fig 5:**
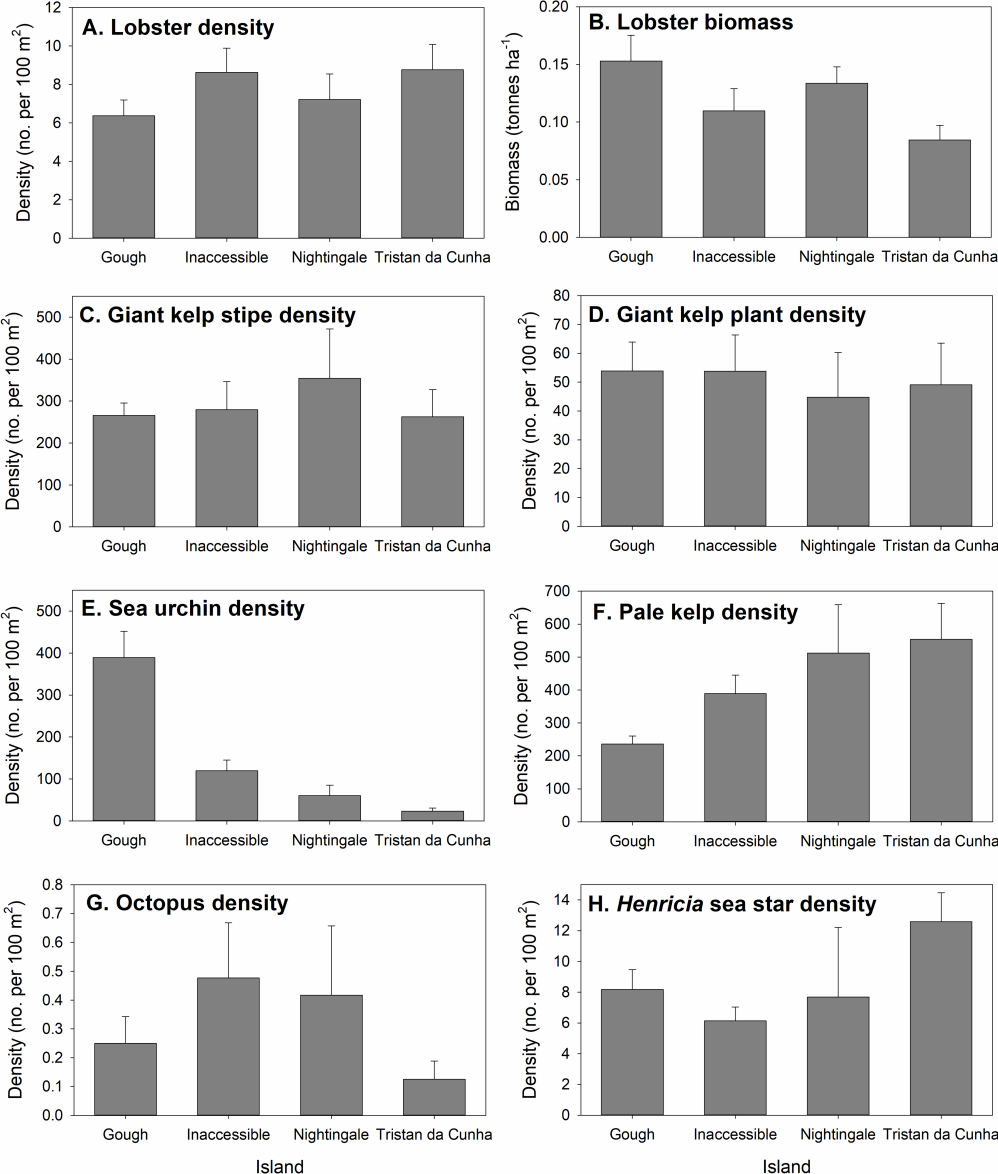
Invertebrate and algal biomass and density-island means. Bar plots depicting island-level variation in lobster biomass and the density of common invertebrates and macroalgae in the Tristan da Cunha Islands from SCUBA surveys of nearshore habitats. Shown are mean values for each species ± 1 standard error of the mean.

**Fig 6:**
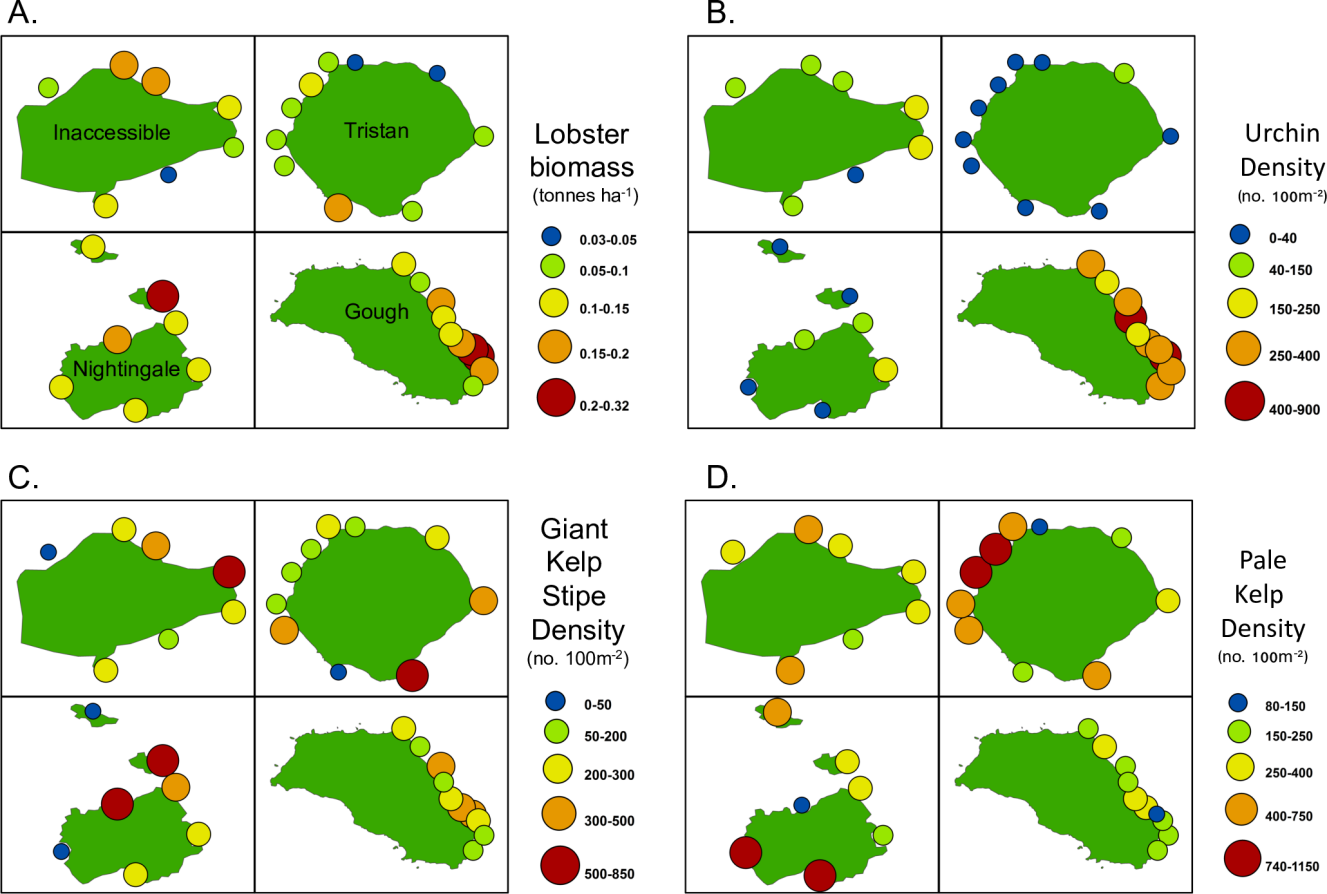
Invertebrate and algal biomass and density-site means. Bubble plots depicting site-level variation in (A) lobster biomass and (B) sea urchin density, (C) giant kelp stipe density, and (D) pale kelp density across the four Tristan da Cunha Islands. Bubble size scales with the biomass or density estimated using site-level means of SCUBA surveys in nearshore habitats.

Lobster (*Jasus tristani*) biomass and density both varied significantly among sites within islands and depth zone (Table 3, S2 Table). Interestingly, the density and biomass patterns were inversely related across islands (Fig 5A and 5B). For example, lobsters were most numerous at Tristan and Inaccessible islands but biomass was highest at Gough and Nightingale. This was due to differences in size structure (S1 Fig), with larger lobsters at Gough (mean size 8.1 ± 0.09 mm carapace length [CL]) and Nightingale (7.5 ± 0.11 mm CL) and smaller lobsters at Tristan (6.6 ± 0.07 mm CL) and Inaccessible (6.7 ± 0.09 mm CL). The variation in biomass among sites within islands was smallest at Nightingale (mean site biomass ranged from 0.10 to 0.21 tonnes ha^-1^) and was largest at Gough (mean site biomass varied from 0.07 to 0.31 tonnes ha^-1^, Fig 6A). Sea urchins (*Arbacia dufresnii*) also varied significantly among islands but there was no effect of site and a weak effect of depth zone (Table 3, Figs 5 & 6). Urchin density was highest at Gough and lower at Inaccessible, Nightingale and Tristan, respectively (Fig 5E, Fig 6B). Other invertebrate species were relatively rare (S2 Table). *Henricia spp*. and octopus densities varied significantly among sites within island and depth zones (Table 3, Fig 5). Octopus were most abundant at Inaccessible and Nightingale, while *Henricia* sea stars were most abundant at Tristan da Cunha. For these two species, we did not observe consistent differences between the northern island group and Gough. Giant kelp (stipes and plants) and pale kelp showed no differences among islands but varied significantly among sites and depth zones.

**Table 3:** Linear mixed models testing the effects of island, site, and depth zone on patterns of invertebrate and macroalgal density and lobster biomass among the Tristan da Cunha islands. Density and biomass were square root transformed. Statistically significant p-values are in bold text. Density and biomass were square root transformed. Presented for the random effect of site are Wald p-values and percent of total variance explained.

	Model r^2^	Factors	df	F-ratio	P-value	Variance explained %
**A. Lobster biomass (*Jasus tristani*)**						
	0.62	Island	3	2.59	0.0711	
		Site[Island]			**0.0341**	**24.8**
		Zone[Site, Island]	34	1.72	**0.0299**	
**B. Lobster density (*Jasus tristani*)**						
	0.69	Island	3	1.35	0.28	
		Site[Island]			**0.0066**	**38.5**
		Zone[Site, Island]	34	1.82	**0.0182**	
**C. Sea urchin density (*Arbacia dufresnii*)**						
	0.87	Island	3	37.50	**<0.0001**	
		Site[Island]			0.0078	36.9
		Zone[Site, Island]	34	1.85	**0.0158**	
**D. *S*ea star density (*Henricia simplex*)**						
	0.78	Island	3	11.34	0.28	
		Site[Island]			**0.0025**	**48.2**
		Zone[Site, Island]	34	3.20	**<0.0001**	
**E. Octopus density (*Octopus vulgaris*)**						
	0.69	Island	3	1.36	0.27	
		Site[Island]			**0.0252**	**27.1**
		Zone[Site, Island]	34	3.18	**<0.0001**	
**F. Giant kelp plant density (*Macrocystis pyrifera*)**						
	0.82	Island	3	0.24	0.87	
		Site[Island]			**0.0013**	**55.6**
		Zone[Site, Island]	34	4.26	**<0.0001**	
**G. Giant kelp stipe density (*Macrocystis pyrifera*)**						
	0.82	Island	3	0.30	0.82	
		Site[Island]			**0.0011**	**57.5**
		Zone[Site, Island]	34	4.22	**<0.001**	
**H. Pale kelp density (*Laminaria pallida*)**						
	0.76	Island	3	2.12	0.1191	
		Site[Island]			**0.0028**	**47.2**
		Zone[Site, Island]	34	2.34	**0.0014**	

#### Community structure and species interactions

**Fig 7:**
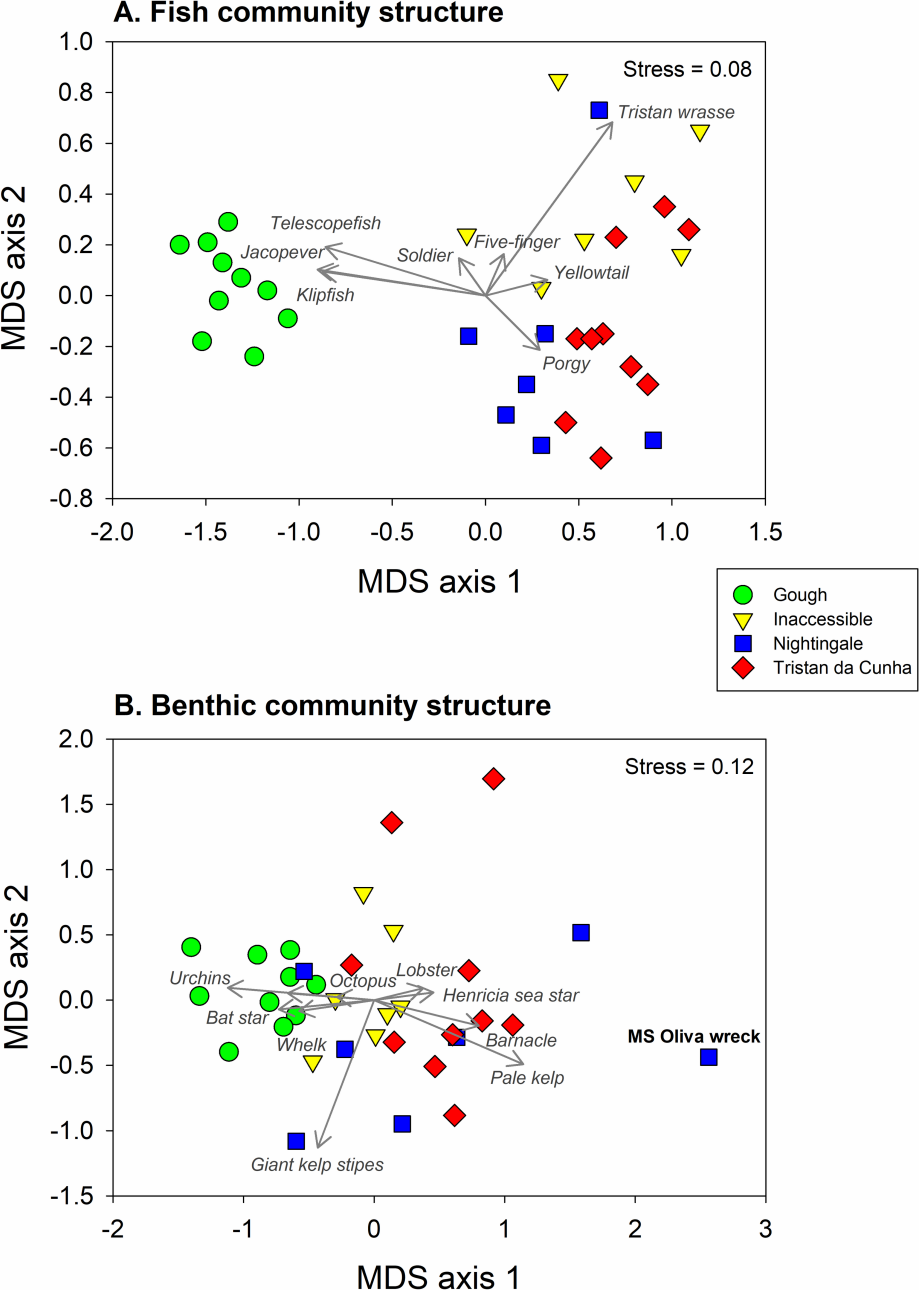
Fish and benthic community structure. Multivariate description of fish and benthic communities in the Tristan da Cunha Islands from nearshore SCUBA surveys. Plots depict non-metric multidimensional scaling (nMDS) analyses of (A) fish assemblages and (B) benthic assemblages using site-level densities of species observed. Vectors overlaying the plot depict the species that are driving separation among sites and islands in species composition in fish and benthic communities. Data were square root transformed prior to analysis.

For fish communities, we found significant differences in assemblage composition among the islands (Table 4, Fig 7). Percentage similarity was highest between the northern island pairs (ranging from 68.3 to 72.8%) while the similarity of Gough to each of the northern islands was much less (ranging from 41.1 to 48.5%). Within islands, site to site variation in fish community structure was quite high and ranged from 75.4% to 86.1%. The differences in fish communities were driven by higher abundances of false jacopever, telescopefish, and klipfish at Gough, but higher abundances of yellowtail, the invasive porgy, and the Tristan wrasse at the northern islands (Fig 7A). SIMPER analyses showing the average percent dissimilarity in fish community structure among pairs of islands and the percent contribution of particular fish species to community dissimilarity are shown in S3 Table. For benthic communities (invertebrates and algae) there was also a significant difference between islands in assemblage composition (Table 5), that was largely driven by differences between Gough and all the northern islands (Fig 7B), similar to our findings for fishes. Percentage similarity was high between the northern island pairs (ranging from 71.6 to 76.9%), while the similarity of Gough to each of the northern islands was less but still higher than for fishes (ranging from 67.3 to 78.9%) (Table 5). Within islands, site-to-site variation in benthic community structure ranged from 65.8% to 85.8%. The primary drivers of island-scale variability in benthic community structure were urchins and the pale kelp. Sea urchins were more abundant at Gough, while the pale kelp was more abundant at the northern islands (Fig 7). A few invertebrate species, the bat star (*Odontaster penicillatus*) and small pink urchin (*Pseudechinus magellanicus*) were only observed at Gough. In contrast, lobsters and *Henricia* were more abundant at the northern islands than Gough. SIMPER analyses showing the average percent dissimilarity in benthic community structure among pairs of islands and the percent contribution of particular invertebrate and algal species to community dissimilarity are shown in S4 Table.

**Table 4:** Results of a PERMANOVA testing differences in fish community structure among the Tristan da Cunha Islands.

**A. PERMANOVA Results**					
**Source**	**df**	**SS**	**MS**	**Pseudo-F**	**P-value**
Island	3	21869.0	7289.5	28.61	0.001
Error	30	7642.5	254.75		
Total	33	29511.0			
**B. Post-hoc pairwise comparisons**					
**Groups**				**t**	**P-value**
Gough vs. Inaccessible				7.73	0.001
Gough vs. Nightingale				6.72	0.002
Gough vs. Tristan				9.11	0.001
Inaccessible vs. Nightingale				2.19	0.018
Inaccessible vs. Tristan				2.05	0.012
Nightingale vs. Tristan				1.78	0.021
**C. Average percent similarity between/within Islands**					
	**Gough**	**Inaccessible**	**Nightingale**	**Tristan**	
**Gough**	86.1				
**Inaccessible**	43.8	75.8			
**Nightingale**	48.5	68.3	75.4		
**Tristan**	41.1	72.5	72.8	77.8	

**Table 5:** Results of a PERMANOVA testing differences in benthic invertebrate and kelp community structure among the Tristan da Cunha Islands.

**A. PERMANOVA Results**					
**Source**	**df**	**SS**	**MS**	**Pseudo-F**	**P-value**
Island	3	4165.3	1388.4	4.57	0.001
Error	30	9122.7	304.1		
Total	33	13288.0			
**B. Post-hoc pairwise comparisons**					
**Groups**				**t**	**P-value**
Gough vs. Inaccessible				2.86	0.001
Gough vs. Nightingale				2.64	0.001
Gough vs. Tristan				3.84	0.001
Inaccessible vs. Nightingale				0.68	0.72
Inaccessible vs. Tristan				1.61	0.046
Nightingale vs. Tristan				0.56	0.85
**C. Average percent similarity between/within Islands**					
	**Gough**	**Inaccessible**	**Nightingale**	**Tristan**	
**Gough**	85.8				
**Inaccessible**	78.9	83.3			
**Nightingale**	67.3	74.6	65.8		
**Tristan**	68.5	76.9	71.6	76.3	

Surprisingly, we found no relationship between giant kelp (primary producer) and urchins (grazer) but a strong inverse relationship between pale kelp and urchins (Figs 5 & 6). During survey and non-survey SCUBA dives, we never observed urchins grazing or resting on giant kelp but we did observe urchins both on the holdfasts and blades of the pale kelp. Lobster density and biomass also showed no clear relationship with urchins (their putative prey) or octopus (their putative predators) but did show similarly low levels of variation among islands as giant kelp.

#### Shipwreck site

We surveyed the shipwreck site of the M/V *Oliva*, which occurred on the western end of Nightingale in 2011 [39]. Surveys took place across much of the structure of the wreck. In general, we saw no dramatic differences relative to other sites at Nightingale (apart from the structure of the wreck itself). Most of the remaining structure is covered completely with benthic turfing and foliose algae and pale kelp (*L*. *pallida*). There was very little giant kelp (*M*. *pyrifera*) at that site, which was situated on the most wave exposed side of the island. In community structure analysis of the benthic community, this particular location was separated in space from all of the other sites, and fell nearer to Tristan da Cunha benthic communities. This result was due to high abundance of barnacles (common on the structure of the wreck) and pale kelp and very low abundance of giant kelp and urchins. For fish communities, no strong differences were observed, likely due to the mobility of this taxonomic group.

### Pelagic camera surveys

Mid-water baited remote underwater video systems (BRUVS) recorded 402 individual pelagic fishes, marine mammals, birds and turtles, representing 13 species from 11 families. Across all sites, mean total abundance per sample set was 11.8 ± 4.21 (SE) individuals. Mean species richness per sample set was 1.7 ± 0.14 (SE) species. The most abundant species was a horse mackerel (*Trachurus* sp. likely *T*. *longimanus*) with 198 individuals observed (MaxN) across 44.4% of sites (S5 Table, S3 Fig). The second most abundant species was yellowtail amberjack (*Seriola lalandi*) with 69 individuals observed, although this species was observed primarily in large schools and thus was only present across 7.4% of sites, all of which were in the northern islands. Blue sharks (*Prionace glauca*) by contrast were observed at 55.6% of sites both in the northern group and at Gough Island, with 23 individuals observed. Yellowfin tuna (*Thunnus albacares*) were observed at 11.1% of sites with 3 individuals noted, all around the northern islands. Observations of all remaining species consisted of one or two individuals occurring at single sites only, this included one sub-Antarctic fur seal (*Arctocephalus tropicalis*) at Gough Island, one loggerhead turtle (*Caretta caretta*), one porbeagle shark (*Lamna nasus*), one pilot fish (*Naucrates ductor*), one crested bellowfish (*Notopogon lilliei*), one oval driftfish (*Schedophilus velaini*), one striped marlin (*Kajikia albida*) and two albacore (*Thunnus alalunga*) at Tristan da Cunha, and two Shepherd’s beaked whales (*Tasmacetus shepherdi*) off Inaccessible Island (S3 Fig).

**Fig 8:**
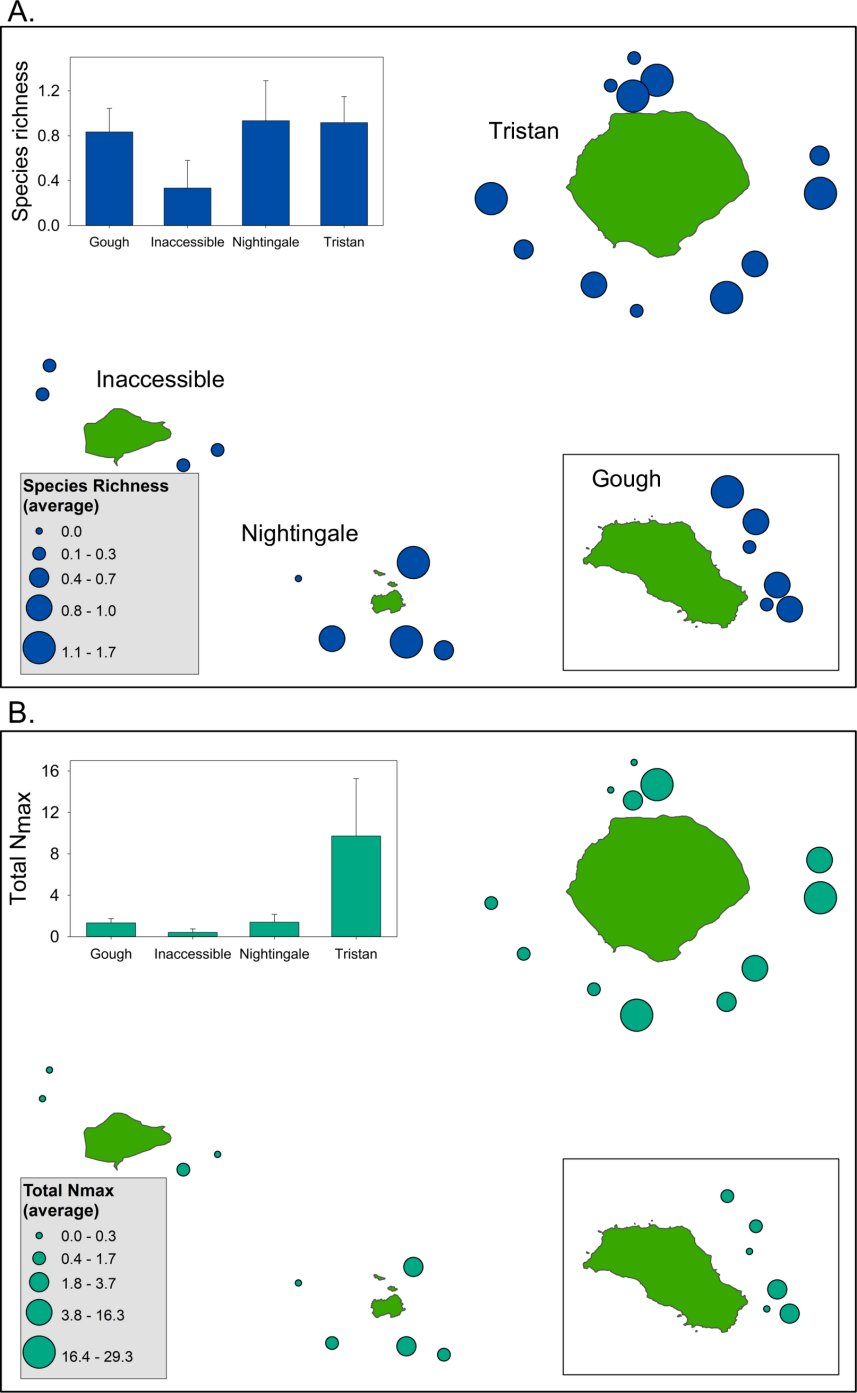
Species richness and abundance from pelagic BRUVs. Bubble plots depicting A. site-level variation in species richness and B. numerical abundance from pelagic BRUV stations sampled across the four Tristan da Cunha Islands. Bubble size scales with the richness or total abundance of individual organisms observed from each pelagic BRUV deployment. Inset bar plots show mean values for each island ± 1 standard error of the mean.

Mean total abundance (summed over all species) differed significantly among islands (N = 27, df = 3, p = 0.049; PERMANOVA, Fig 8) driven by the high total abundance at Tristan compared to the other islands. Species richness did not differ among islands (N = 27, df = 3, p = 0.171; PERMANOVA, Fig 8).

**Fig 9:**
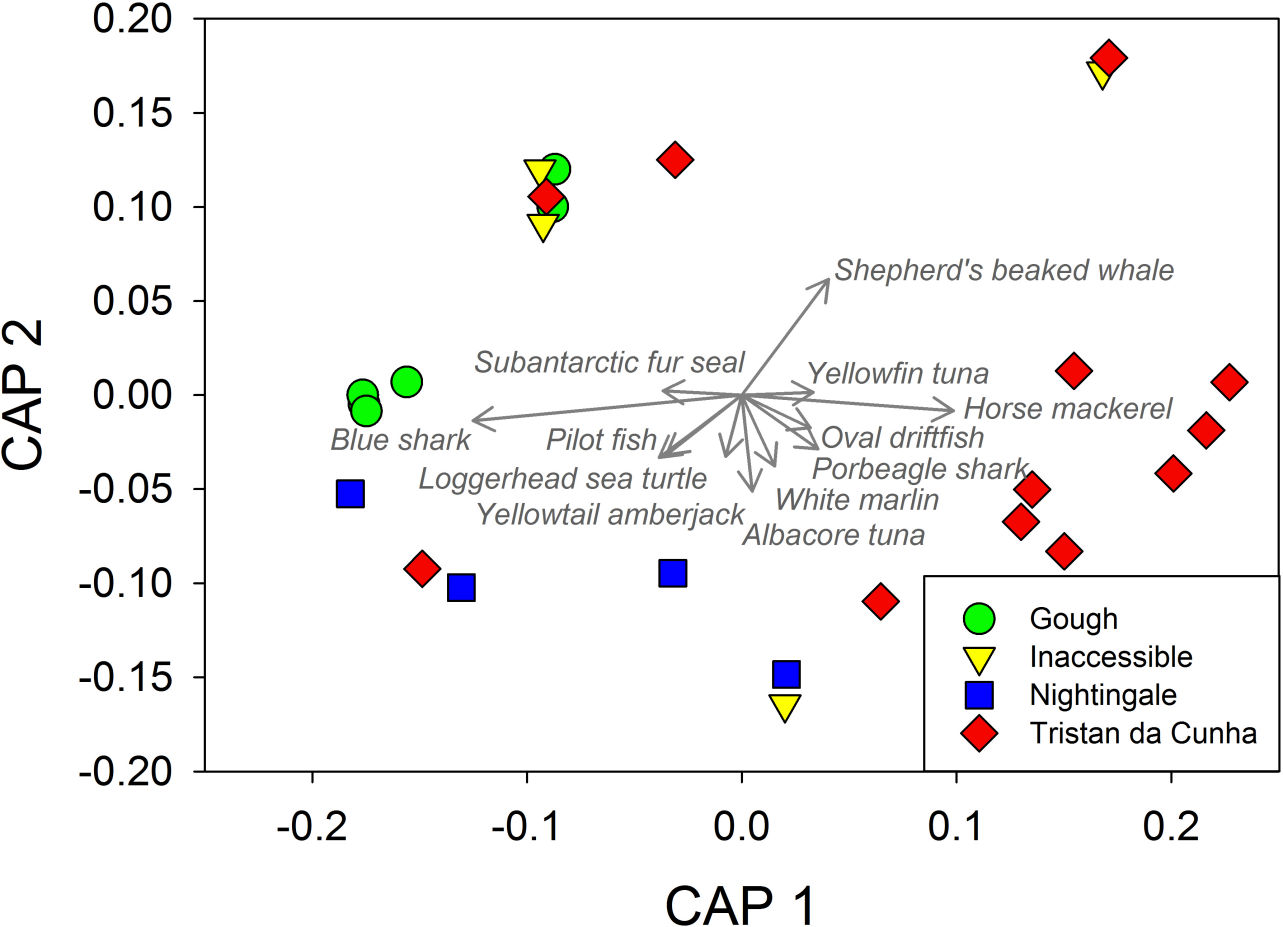
Multivariate description of pelagic assemblages in the Tristan da Cunha islands from mid-water BRUVS surveys. Plots depict canonical analysis of principal coordinates (CAP) analyses based on a Bray-Curtis resemblance matrix site-level species specific abundances. Vectors overlaying the plot depict the species that are driving separation among sites and islands in species composition. Data were square root transformed prior to analysis.

There was also significant island-to-island variation in pelagic assemblages characterized by individual species abundances (Table 6). Pairwise comparisons revealed significant differences in assemblage structure between Tristan and Gough, and Nightingale and Gough (Table 6) indicating that the northern islands have a different species composition to Gough. A CAP plot of pelagic species abundance by site (Fig 9) indicated that all sites at Gough were extremely similar to one another and that the primary species driving differences in assemblages among sites were blue sharks with more found at Gough and horse mackerel which tended to be more abundant at Tristan.

**Table 6:** Results of a PERMANOVA testing differences in pelagic community structure among islands from the mid-water BRUVS surveys at the Tristan da Cunha Islands. Data are species abundances (MaxN) and were square root transformed.

**PERMANOVA Results–square root transformed data**					
**Source**	**df**	**SS**	**MS**	**Pseudo-F**	**P-value**
Island	3	21782	7260.8	3.1425	0.006
Error	20	46211	2310.5		
Total	23	67993			
**Post-hoc pairwise comparisons**					
**Groups**				**t**	**P-value**
Tristan, Nightingale				1.46	0.095
Tristan, Gough				2.92	**0.001**
Tristan, Inaccessible				0.98	0.477
Nightingale, Gough				1.77	**0.017**
Nightingale, Inaccessible				0.39	1
Gough, Inaccessible				1.71	0.181
**Average percent similarity between/within Islands**					
	**Tristan**	**Nightingale**	**Gough**	**Inaccessible**	
**Tristan**	29.3				
**Nightingale**	24.4	35.5			
**Gough**	15.1	43.3	69.4		
**Inaccessible**	21.1	39.5	35.7	0	

In some cases, the camera rigs were left to drift longer than the standardized 2 hr period. This additional footage was scanned for the presence of additional species. This extension yielded 106 additional individuals, new species included a single bluefish (*Hyperoglyphe antarctica*) of 84.8 cm at Nightingale Island, a northern rockhopper penguin (*Eudyptes moseleyi*) of 36.7 cm, and 5 dusky dolphins (*Lagenorhynchus obscurus*) at Gough Island. There is a large population of northern rockhopper penguins on Gough Island and large pods of dusky dolphins were seen at the surface on multiple occasions while sampling on the northern side of the island.

### Deep-water drop camera surveys

Deep water drop cameras were deployed at multiple sites (Fig 1) and in a variety of depths on each island (S1 Table). In all, we completed *n* = 23 deep water drops to depths ranging from 164 m to 1414 m (S1 Table). Cameras were timed to record for 2 hours but several drops suffered premature releases, sometimes due to sharks severing the anchor lines (Drops T9, T17 and T21; S1 Table). We observed a total of 21 species or species groups and frequency of occurrence (occurrence on a drop/total number drops) ranged from 0.04 to 0.61 (S6 Table). Several species or groups were observed on greater than 30% of deep-water drops (S4 Fig). These included: Bluenose (*Hyperoglyphe antarctica*), Lantern sharks (*Etmopyerus granulosus*), Bluntnose sixgill shark (*Hexanchus griseus*), Grenadiers (family Macrouridae), Deepwater cod (*Physiculus karrerae*), and Cutthroat eel (*Synaphobranchus* sp., likely *brevidorsalis*). Shark observations varied, with lantern sharks seen at 56% of drops from 714–1404 m, Bluntnose sixgill sharks on 30% of drops from 190–1027 m and a single Sevengill shark recorded on a drop at 164 m. Not all recorded organisms were fish, a southern elephant seal (*Mirounga leonina*) was observed at a single camera drop at 190 m depth. Habitat varied on the deep-water drops. Observed biotic habitat forming organisms included whip corals, gorgonians, sea pens and sea fans (S4 Fig). The benthos also varied with some drops on sand/mud habitat and others on what appeared to be moderate to high rock relief.

**Fig 10:**
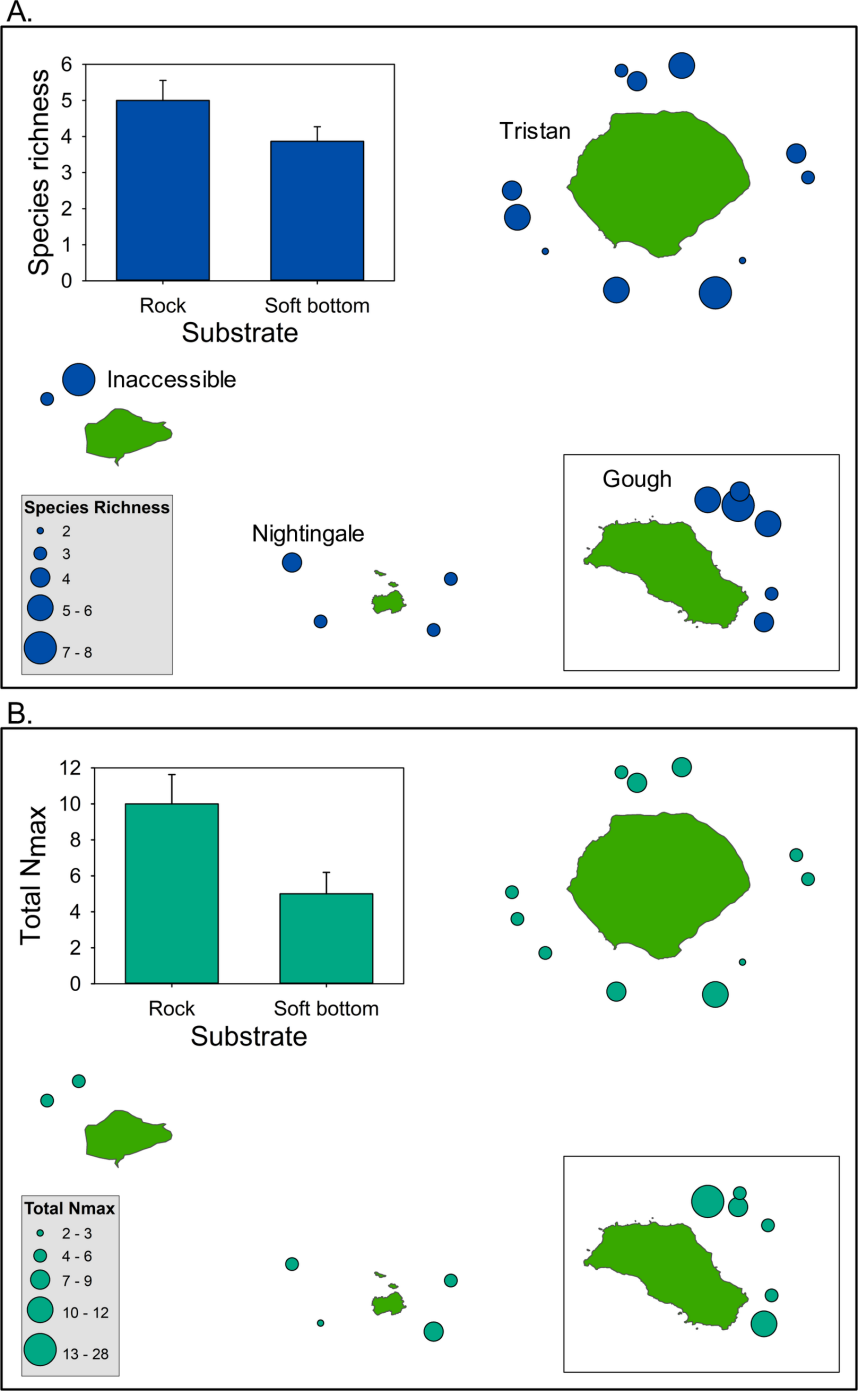
Species richness and abundance from deep water drop cameras. Bubble plots depicting A. site-level variation in species richness and B. numerical abundance from deep-water drop-camera stations sampled across the four Tristan da Cunha Islands. Bubble size scales with the richness or total abundance of individual fishes observed from each drop camera deployment. Inset bar plots show means (± 1 standard error of the mean) for the two benthic types.

**Fig 11:**
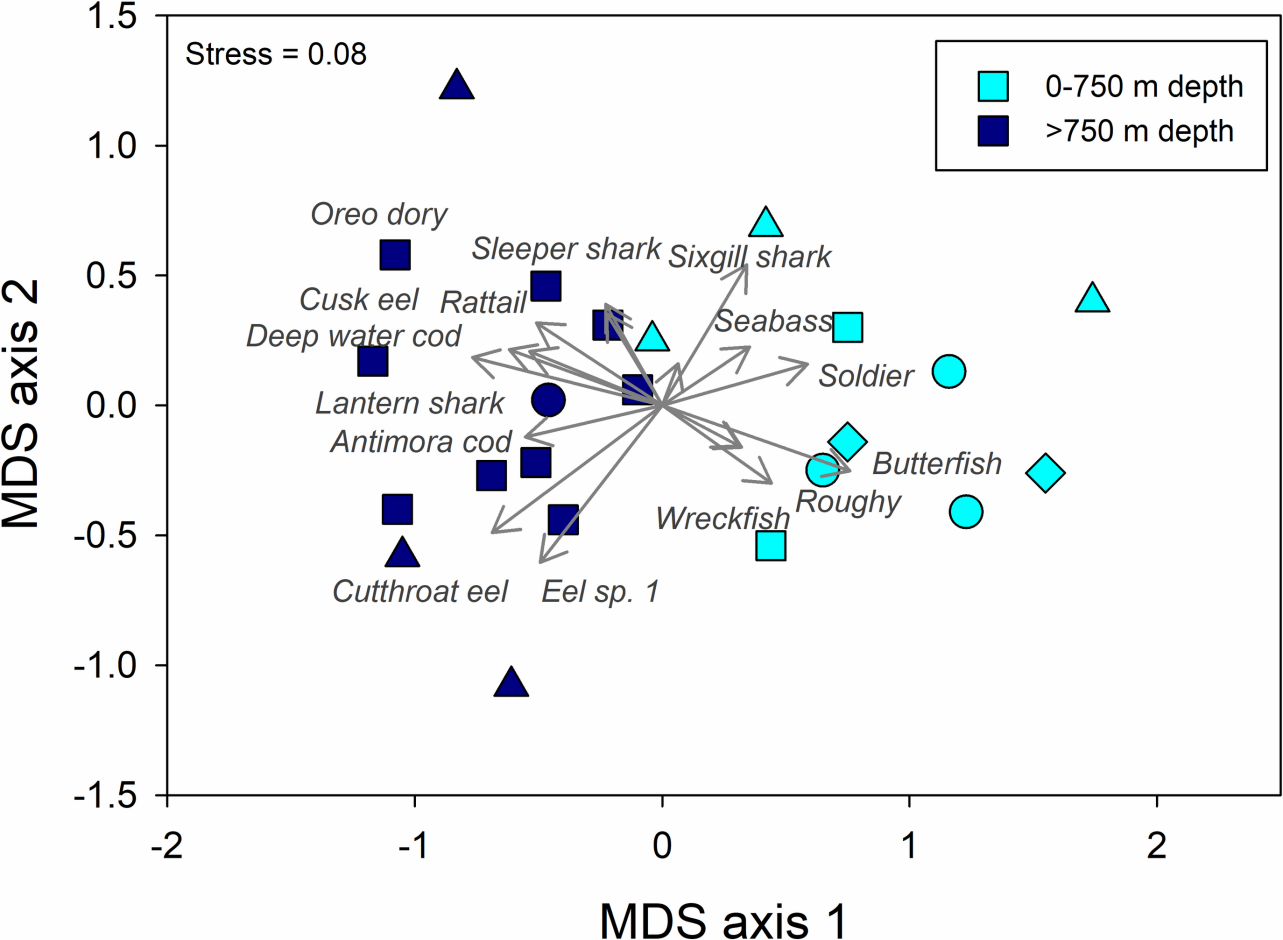
Multivariate description of the fish community in the Tristan da Cunha Islands from deep-water camera surveys. Multivariate description of the fish community categorized into two depth strata (0–750 m and >750 m depth). Shown is a non-metric multidimensional scaling (nMDS) analysis of the fish assemblage using presence/absence data at each station and a Euclidean distance matrix. Vectors overlaying the plot depict the species that are driving separation among sites and islands (Gough = triangle symbols, Inaccessible = diamond symbols, Nightingale = circle symbols, Trista da Cunha = square symbols) in species composition in deep-water fish communities.

Species richness ranged from 2 to 8 species per drop and did not exhibit consistent spatial patterns except that the four drops at Nightingale were all relatively low in species richness (Fig 9A). Total MaxN (sum of MaxN across species in a drop) ranged from 2 to 28 individuals per drop and differed spatially among drop-camera stations (Fig 10B), with the highest fish abundances on Gough and Tristan compared to the other two islands. Drop camera stations were categorized as having primarily soft or rock substrate. We observed a non-significant trend for higher species richness in drops on rocky habitat (t_21_ = 1.64, P = 0.11) and a significant effect of substrate on total MaxN (t_21_ = 2.47, P = 0.022), with more fish individuals observed in drops on rocky habitat. Drop camera stations were also categorized into two different depth zones (0–750 m and >750 m depth) for further analysis of diversity and community patterns. We did not observe significant differences in species richness (t_21_ = 0.93, P = 0.36) or total MaxN (t_21_ = 1.04, P = 0.31) between the drops conducted in the two depth zones. However, we found that the deep-water fish assemblages differed significantly between the shallower and deeper strata, but did not differ among islands (Fig 11, Table 7). Communities shallower than 750 m were characterized by the presence of soldiers (*Heliocolenus mouchezi*), Southern butterfish (*Hyperoglyphe antarctica*), wreckfish (*Polyprion oxygeneios)*, small seabass and roughy (*Lepidoperca coatsii*, *Beryx decadactylus*), while communities deeper than 750 m were composed of deepwater and antimora cods (*Physiculus karrerae*, *Antimora sp*.), cusk eels Unidentified in this study), grenadiers (*Coelorinchus* sp.), cutthroat eels (*Synaphobranchus* sp. (likely *brevidorsalis*)), and lantern sharks. (*Etmopterus* sp. (likely *granulosus*)).

**Table 7:** Results of a PERMANOVA testing differences in fish community structure between depth categories (0–750 m vs. >750 m depth) from the deep-sea drop-cam surveys at the Tristan da Cunha Islands.

A. PERMANOVA Results–Presence/absence data					
Source	df	SS	MS	Pseudo-F	P-value
Depth category	1	15.21	15.2	8.7	0.001
Error	21	36.7	1.7		
Total	22	51.9			

## Discussion

### Overview

Tristan da Cunha Islands are a unique archipelago with healthy marine ecosystems, although species-poor, likely due to the extreme isolation of the four islands from any other landmasses (>2000 km distance) [44]. Despite low levels of biodiversity, the high abundance and biomass of the taxa present indicates that the nearshore ecosystem is healthy and relatively intact. The health of the ecosystem in light of an ongoing commercial rock lobster fishery (which provides 80–90% of the income for the islands) and subsistence fishing for local islanders, indicates that exploitation levels are relatively low and that the resources are being well managed. These remote temperate islands provide one of few remaining places in the world to establish a baseline for an unimpacted temperate system, given the concentration of human populations in coastal temperate zones across the planet. Prior to this expedition, quantitative data describing abundances and biomass of key organisms in nearshore kelp forests was lacking, while pelagic and deep sea benthic habitats were mostly unexplored.

### Kelp forests

#### Fish biomass

Although there are few temperate island systems to compare to, and none as isolated as Tristan, we found that levels of fish biomass were comparable to remote islands in Chile as well as to less remote islands in California, USA. Both locations are broadly similar to Tristan in habitat (i.e. kelps as the dominant biotic habitat former) and sea temperature, and both are locations with spiny lobster fisheries. Total fish biomass across the Tristan da Cunha islands ranged from 1.5 to 2.75 t ha^-1^ compared with an average of 2.3 t ha^-1^ at the remote Desventuradas and Juan Fernandez islands in Chile [45]. The northern Channel Islands (NCI) in California is an area with a large amount of available long term monitoring data [40, 46] and despite very large differences in fish species richness (2–4 species per site in Tristan da Cunha and 11–22 per site in the NCI), total fish biomass in the Tristan Islands is comparable to and in many cases higher than most sites in the NCI. Thus, for the few species observed at Tristan, abundance and biomass is extremely high. The average fish biomass across the NCI locations was 1.2 t ha^-1^. The highest recently recorded fish biomass for the Channel Islands was 2.7 t ha^-1^ and this was inside a marine reserve that restricted fishing for over 10 years [46]. Other temperate and tropical locations have recorded biomass estimates from as low as 0.4 t ha^-1^ to as high as 4.5 t ha^-1.^ The variation is related to levels of fishing pressure and abundance of large bodied sharks and other top predators [13, 47, 48].

#### Lobster density and biomass

Lobster density was also extremely high at the Tristan da Cunha Islands with a mean of 6.4 to 8.8 lobsters per 100 m^2^ across the islands, compared to an average density of rock lobsters in the NCI (a different species to Tristan, *Panularis interruptus*) of 0.66 per 100 m^2^. In California, the highest density of lobster ever observed on an annual survey over 15 years of monitoring was 9.1 lobsters per 100 m^2^ and this occurred inside a marine reserve that has been protected from fishing for over 30 years (Caselle unpub. data). Despite being fished at Tristan da Cunha, lobster abundances there are comparable to densities of lobsters in protected areas in other parts of the world. For example, mean densities of the spiny lobster, *Panulirus cygnus*, in protected areas in Rottnest Island, West Australia were 7.08 lobsters per 100 m^2^ (compared to 0.2 per 100 m^2^ in fished areas) [49] and the highest densities of *Jasus edwardsii* in multiple protected areas across New Zealand were 6 and 12 lobsters per 100 m^2^ in two MPAs that were protected for roughly 15 years [50]. While lobster densities and biomass at Tristan da Cunha differed little among islands, there were some important within-island variability. Notably, the lowest biomass was observed closest to the harbor and settlement on Tristan, indicating the potential for some local depletion due to fishing pressure in proximity to port. Lobsters also exhibited spatial differences in size structure, with the largest lobsters occurring in the colder waters of Gough, similar to previous reports [20] and fisheries-dependent data. Anecdotally, during the expedition several small lobster pots were set at Tristan for personal consumption and we observed 20–40 lobsters caught per trap with soak times as little at 3 hours. Our experience in California, where lobster fishing pressure is much more intense, is that even larger traps set for several days would not catch that density of lobsters. However, it should also be noted that the same duration trap sets at Gough during the expedition all came up empty or with lower numbers, indicating some variance in CPUE across islands. We saw no evidence for density dependent interactions with lobster, in fact, settlement of this species in the Tristan islands is hypothesized to occur in shallow rockpools and intertidal areas, and not likely overlapping with the adult distribution.

#### Giant kelp

Giant kelp, *Macrocystis pyrifera* was the major habitat forming species across the Tristan Islands, with the pale kelp, *Laminaria pallida* forming dense sub-canopy forests at some sites. Interestingly, giant kelp plants across the Tristan islands were dominated by small (median = 2–5 stipes per plant across the four islands; S1 Fig) individuals, compared to other regions of the world where each plant can be massive, often containing 100's of individual stipes [51, 52]. For example, in another south Atlantic island of the Falklands, stipes ranged from 67–109 per plant [53] while *Macrocystis* in California range from 10–15 stipes per plant on average although stipe counts of >100 are frequently found [54]. Stipe number is a measure of growth in *Macrocystis* and is sensitive to temperature, depth, latitude, degree of wave exposure, upwelling, and other factors [55–57]. Divers also observed that the giant kelp at Tristan appeared to be extremely tough, with blades that resisted tearing, very thick stipes and numerous floats. Sea conditions at Tristan da Cunha are notoriously rough; storms, large waves and very high winds are common (often limiting fishing opportunities to ~60 days per year) and it is likely that *M*. *pyrifera* suffer frequent high mortality events. It has been suggested that plants with low stipe counts might have higher survivorship due to a lowered rate of entanglement and dislodgement [58]. California’s kelp forests have demonstrated high turnover rates, with standing crops estimated to regenerate upwards of seven times per year [51], and high rates and intensity of disturbance at Tristan da Cunha likely cause even higher rates of turnover. The small size and young age of the kelp plants likely reflects the high probability of dislodgement for larger plants that experience higher drag forces [59, 60]. Giant kelp at Tristan da Cunha are potentially locally adapted to the inclement sea conditions experienced year round, but this remains to be further investigated through comparative biomechanic and genetic studies. The consequences of this growth form for resilience to climate change-induced effects (which may include increased sea temperatures or increases in waves and sea state) are currently unknown.

#### Spatial variation among and within islands

We found large differences in kelp forest assemblage structure between the southern island of Gough and the three northern islands. The differentiation was stronger for fish but also existed for the benthic invertebrate and algal communities. The differences in both species composition and size structure between Gough and northern islands is likely driven by differences in temperature and oceanographic productivity above and below the subtropical convergence zone (STC). The STC is a major feature of the Tristan’s offshore waters and is normally located between the northern islands and Gough, with Gough sitting somewhere in the frontal region. The STC delimits the northern limit of the Antarctic Circumpolar Current [61] and corresponds to the boundary of the Southern Ocean (following [62]). The STC is generally characterized by a surface- temperature discontinuity of 4 or 5°C and a salinity difference of 0.5 x 10^−3^ [24] with warmer, more saline waters north of the front, partly derived from strong southward currents. Our oceanographic sampling confirmed a strong temperature and salinity difference somewhere between the northern islands and Gough. Surface water temperature ranged from 14.9°C at Gough to 18.6°C at Tristan da Cunha while surface salinity ranged from 34.57 PSU at Gough to 35.23 PSU at Tristan da Cunha. Colder temperature and higher productivity lead to faster growth rates, potentially explaining the larger fish and lobsters at Gough relative to the other islands. However, lower fishing pressure at Gough may also contribute to the presence of larger animals at that island.

Within each island, we found a remarkable lack of variation in measured habitat features such as benthic substrate and physical relief (S2 Fig). Although we focused on rocky reefs that had emergent kelp canopy and avoided large expanses of volcanic sand (which do exist between reefs), almost all sites surveyed were composed of moderate or very large sized boulders and very low cover of sand or cobbles. Even within our coarse categories of physical relief, the average relief across islands was similar (S2 Fig). There are virtually no flat areas on these reefs. These boulder reefs provided ample crevices and large overhangs that were utilized extensively by lobsters. We also found very similar island-wide averages of kelp plant and stipe density, despite large differences in temperature between the northern islands and Gough. We hypothesized that Gough would have thicker, healthier kelp beds due to exposure to colder and more nutrient rich waters below the subtropical convergence [23, 63], but this was not found. It is likely that the primary driver of kelp abundance in this system is physical stress due to chronic, year round wave and storm impacts [64, 65].

The lack of biodiversity in the kelp forests of Tristan results in a very simple food web with limited ecological interactions. While detailed feeding habits and information for the key species in Tristan's kelp forests are unknown at this time, we can hypothesize the linkages based on known prey preferences for these taxa in other locations [38, 44, 66–72]. Our surveys quantified the key components of this simple food web, with kelps at the base providing both food and biophysical habitat; urchins (and possibly the invasive silver porgy) as the primary grazers; telescopefish as likely planktivores; five-finger, wrasses, and lobsters as omnivores; octopus and false jacopever as carnivores; and sevengill sharks and possibly yellowtail as the primary piscivores. Additional species that may occasionally feed in the kelp forest include subantarctic fur seals and rockhopper penguins. Simple food webs such as Tristan's may be less resilient in the face of perturbations that might reduce or remove one of the key trophic links than systems with greater functional redundancy [73, 74]. The extreme isolation of the Tristan da Cunha likely prevents colonization via dispersal and species turnover or changes in dominance are less likely than in more highly connected systems. We identified two key members of this food web that might be at risk. Some areas of the world have experienced massive declines in giant kelp (*M*. *pyrifera*) [75, 76], (but see [77, 78]) and in Tristan's northern islands this species may currently be at or near its upper thermal limits. Future increases in temperature could place this species at risk with unknown but likely dramatic effects on the entire ecosystem. Pale kelp (*L*. *pallida*), while abundant and more resilient to higher sea temperatures, might increase but its role as a potential additional foundation species in the ecosystem is unknown. We observed urchins grazing only on pale kelp, not on giant kelp, but lobsters have been observed to consume both pale kelp and giant kelp [44]. Lobsters are the other key species that may be at risk from both fishery effects and climate change. Currently the lobster fishery appears extremely well managed, with conservative annual quotas [20]. However, many important life-history attributes such as recruitment and growth rates are not known and could be affected by climate related changes, either directly or through indirect responses to changes in the ecosystem or prey base. Lobster are the purported predators on urchins in this system and it has been demonstrated in many temperate systems that the loss of this key species can transition systems to urchin barrens [79–82]. For simple food webs with little functional redundancy and hence resilience, it is critical to ensure that the key players remain at healthy levels of abundance [83, 84].

#### Shipwreck site

Nightingale Island suffered the wreck of the bulk carrier MS Oliva in March 2011, which ran aground on the western end of the island. The subsequent break-up of the vessel contaminated Nightingale and nearby Inaccessible with 1500 t of heavy fuel oil, depositing 65,000 t of soya beans on the seabed around Nightingale. Our surveys of the wreck indicated that fish and lobster biomass were low at the wreck site itself and the benthic community differed, with high barnacle abundance and no giant kelp. Otherwise, the surrounding area was typical of a highly wave exposed site in this region. At the island-scale, Nightingale currently has the highest total fish biomass and the second highest lobster biomass, indicating that direct effects of the wreck were localized and that the marine life has begun to recover.

### Pelagic and deep-sea habitats

The subtropical convergence zone around the Tristan da Cunha islands is a hotspot for pelagic diversity in the Atlantic Ocean, likely due to high productivity of these oceanic fronts. Recent work has demonstrated the importance of oceanographic features such as fronts to apex predators via enhanced primary and secondary productivity [28, 85]. The Tristan islands are a globally recognized region for seabirds and also home to pelagic sharks, tunas, cetaceans and seals. During our expedition, we documented many of these offshore roaming marine species on our pelagic cameras including blue and porbeagle sharks; tunas, yellowtail and marlin; dolphins; fur seals, turtles and the rare Shepard’s beaked whale. The extent to which these species and the seabirds utilize the STC as a critical feeding area is unknown at this time but in other regions, these areas are important for population persistence [27, 29, 86]. The pelagic cameras recorded an average species richness of 1.7 species per drop and average abundance (measured as MaxN) of 11.8 individuals. Notably, blue sharks were extremely common in the pelagic zone, occurring on just over half of camera deployments. Surprisingly, we recorded a relatively high abundance of small juvenile and likely newborn blue sharks, indicating that the islands may serve as a pelagic nursery for this species. Observations of a juvenile porbeagle shark and Shepherd’s beaked whales are the first underwater documentation of these species in the islands.

This expedition provided one of the first surveys of very deep habitats around Tristan (but see [24] for list of all prior work, including deep water scientific trawls). The deep-sea drop-cameras indicated that species richness and abundance is positively related to the presence of hard rocky substrate (present on 35% of camera drops). Biogenic habitats were present at the deep-sea camera sites on 40% of the deployments, including sea pens, crinoids, whip corals, and small to very large gorgonians. Many of the taxa observed in the deep-sea off Tristan da Cunha also occur in other locations around the globe where these camera systems have been deployed [47, 87], indicating some level of uniformity in deep-sea fish communities. However, we did observe distinct differences in the fish community above and below 750 m depth. Our oceanographic sampling also showed a discontinuity or ‘breakpoint’ in temperature and salinity at approximately 600–750 m depth. At this depth range, we found that both variation among islands and total variation became minimal with further increases in depth Although levels of dissolved oxygen with depth showed more complex patterns, it was at this same depth range where among island variation was reduced. While our CTD measurements were a snapshot in time, this dataset is the first to our knowledge in the nearshore areas of Tristan Islands. Further work will be needed to understand the effects of ocean conditions on deepwater assemblages, however here we have identified depth zones between which both assemblage structure and oceanography showed marked differences.

### Conservation and management recommendations

The Tristan da Cunha islands are a unique archipelago with healthy marine ecosystems–although with low species diversity, likely due to extreme isolation [44]. This remote temperate archipelago provides one of the few places in the world to establish a baseline for unimpacted temperate systems. Quantitative data from the kelp forests was lacking and pelagic habitat and deep benthos were mostly unexplored prior to this expedition. We found that, despite an important commercial fishery for lobster and subsistence fishing for local islanders, marine habitats and biota appeared in very good condition. Biomass of fishes and lobster in particular were high. However, this unique marine ecosystem is not without potential threats: shipping traffic leading to wrecks and species introductions, pressure to increase fishing effort beyond sustainable levels and the impacts of climate change all could potentially increase in the coming years. Currently, the low population density, difficult access to the marine environment and a pro-active, well-managed lobster fishery provide a level of protection to nearshore habitats. However, offshore areas including seamounts, would benefit from strong, enduring protection, especially from illegal, unlicensed and unregulated fishing activity.

The Tristan da Cunha Exclusive Economic Zone (EEZ) spans just over 3° latitude and contains approx. 754,000 km^2^. Due to the unique placement of the islands in the Southern ocean the EEZ also includes a range of oceanographic features and offshore habitats such as seamounts. The Tristan Islands are a globally recognized region for seabirds and also home to pelagic sharks, tunas, cetaceans, and seals. Recent work has demonstrated the importance of oceanographic features such as fronts to apex predators via enhanced primary and secondary productivity [28, 85]. Unfortunately, fronts and convergences are also areas with high fishing pressure, primarily from longline vessels. Longline fishing is responsible for the deaths of an astonishing number of seabirds annually [88] and interactions between pelagic sharks and longline vessels is common [34]. While there is currently little to no longline fishing in Tristan’s waters, protection of these offshore habitats (such as convergence zones and seamounts) would likely add significant future insurance for a large number of important species that utilize them.

Deep sea habitats around the Tristan islands were diverse with many sites having biogenic coverage on rocky substrates that provided habitat for fishes. These deep biogenic habitats are extremely vulnerable to disturbance from trawl gear and future management should geared towards limiting bottom contract with trawl operations at offshore seamounts. Our drop camera surveys only scratched the surface of deep water exploration in the region and we were, regrettably, not able to survey the many seamounts in the EEZ. Deep water biodiversity and assessment of the vulnerability of these habitats should be the focus of future research and monitoring efforts.

The United Kingdom has committed to protection of over four million square kilometers of marine environment across the UK Overseas Territories, including Tristan da Cunha. The Blue Belt program aims to support the commitment by providing scientific information and guidance on management strategies including enforcement and surveillance (https://www.gov.uk/government/publications/the-blue-belt-programme). Our surveys and the results presented here can be used to inform future management decisions as well as provide a baseline against which future monitoring can be based.

## Supporting information

S1 Fig Size structure from SCUBA surveys. Spatial variation in size structure of lobsters, giant kelp, and two common nearshore fish species estimated using visual SCUBA surveys. Shown are size frequency histograms for each species pooled at the island-level. Arrows above the histogram depict the estimated mean size at each island. Values on plots are the mean size ± 1 standard error of the mean.
(PDF)

S2 Fig Substrate and habitat relief. Substrate composition and habitat complexity estimated using visual SCUBA surveys in nearshore kelp forests (10 and 20 m depth) in the Tristan da Cunha Islands. Shown are the percent cover of (A) four different substrate categories and (B) vertical relief in four different relief categories.
(PDF)

S3 Fig Pelagic BRUV species. Photos depicting representative species observed on pelagic camera drops at the Tristan da Cunha Islands. (A) Krill school (Euphausids spp.), Gough; (B) Blue sharks (Prionace glauca.), Nightingale; (C) Recently born blue shark (Prionace glauca.), Tristan da Cunha; (D) Porbeagle shark (Lamnin nasus), Tristan da Cunha; (E) Southern horse mackerel (Trachurus longimanus), Inaccessible; (F) Yellowtail amberjack school (Seriola lalandi), Tristan da Cunha; (G) Striped marlin (Kajikia albida), Tristan da Cunha; (H) Yellowfin tuna (Thunnus albacares), Tristan da Cunha; (I) Loggerhead turtle (Caretta caretta.), Tristan da Cunha; (J) Subantarctic fur seal (Arctocephalus tropicalis), Gough; (K) Dusky dolphin (Lagenorhynchus obscurus), Gough; (L) Shepherd’s beaked whale (Tasmacetus shepherdi); Inaccessible.
(PDF)

S4 Fig Deep sea species. Photos depicting representative fish and habitat-forming benthic species observed on deep-sea camera drops at the Tristan da Cunha Islands. (A) Bluntnose sixgill shark (Hexanchus griseus), (B) Lantern shark (Etmopterus sp., likely granulosus), (C) Cutthroat eel (Synaphobranchus sp., likely brevidorsalis), (D) Deepwater cod (Physiculus karrerae), (E) Roughy (Beryx decadactylus), (F) Oreo dory (Neocyttus sp.), (G) Soldier (Heliocolenus mouchezi) and Octopus (Octopus vulgaris), (H) Southern butterfish (Hyperoglyphe antarctica.), (I) Oval driftfish (Schedophilus velaini), (J) Whip coral, (K) Deepwater gorgonian, (L) Field of gorgonians and crinoids, (M) Sea pens.
(PDF)

S1 Table Deep-sea drop camera deloyments. Details of the deep-sea drop camera deployments for all stations surveyed in the Tristan da Cunha Islands group.
(PDF)

S2 Table Biomass and density from SCUBA surveys. Biomass (tonnes ha-1) and density (no. 100 m2) of fish, conspicuous benthic invertebrates, and kelps observed on SCUBA surveys at 10 and 20 m depth in the Tristan da Cunha Islands. Values are means ± 1 standard error of the mean for each island.
(PDF)

S3 Table Similarity Percentage Analysis (SIMPER) showing the average percent dissimilarity in the fish assemblage structure among the four Tristan da Cunha islands. Shown are the top three species contributing to community dissimilarity on each island and the percent contribution of each species to community dissimilarity in each pair-wise comparison. Mean densities of these and other species by site are shown in S2 Table.
(PDF)

S4 Table Similarity Percentage Analysis (SIMPER) showing the average percent dissimilarity in the benthic community structure among the four Tristan da Cunha islands. Shown are the top three species contributing to community dissimilarity on each island and the percent contribution of each species to community dissimilarity in each pair-wise comparison. Mean densities of these and other species by site are shown in S2 Table.
(PDF)

S5 Table Pelagic BRUV taxa and abundance. Species observed in pelagic BRUVS surveys at Tristan da Cunha group. Total number of individuals recorded using the maximum MaxN per site, and the Mean MaxN per site with associated standard error (SE), and the percentage of sites that species were present.
(PDF)

S6 Table Deep sea fish. Fish taxa observed on deep-sea drop-cams in the Tristan da Cunha Islands group. Freq.–frequency of occurrence out of all drops (n = 23).
(PDF)

## References

[1] Jackson JB, Kirby MX, Berger WH, Bjorndal KA, Botsford LW, Bourque BJ, et al Historical overfishing and the recent collapse of coastal ecosystems. Science. 2001;293(5530):629–37. doi: 10.1126/science.1059199 11474098 10.1126/science.1059199

[2] Lotze HK, Lenihan HS, Bourque BJ, Bradbury RH, Cooke RG, Kay MC, et al Depletion, degradation, and recovery potential of estuaries and coastal seas. Science. 2006;312(5781):1806–9. doi: 10.1126/science.1128035 16794081 10.1126/science.1128035

[3] Pandolfi JM, Bradbury RH, Sala E, Hughes TP, Bjorndal KA, Cooke RG, et al Global trajectories of the long-term decline of coral reef ecosystems. Science. 2003;301(5635):955–8. doi: 10.1126/science.1085706 12920296 10.1126/science.1085706

[4] Worm B, Sandow M, Oschlies A, Lotze HK, Myers RA. Global patterns of predator diversity in the open oceans. Science. 2005;309(5739):1365–9. doi: 10.1126/science.1113399 16051749 10.1126/science.1113399

[5] Bax N, Williamson A, Aguero M, Gonzalez E, Geeves W. Marine invasive alien species: a threat to global biodiversity. Marine Policy. 2003;27(4):313–23. doi: 10.1016/s0308-597x(03)00041-1

[6] Hoegh-Guldberg O, Bruno JF. The impact of climate change on the world’s marine ecosystems. Science. 2010;328(5985):1523–8. doi: 10.1126/science.1189930 20558709 10.1126/science.1189930

[7] Halpern BS, Walbridge S, Selkoe KA, Kappel CV, Micheli F, D'agrosa C, et al A global map of human impact on marine ecosystems. Science. 2008;319(5865):948–52. doi: 10.1126/science.1149345 18276889 10.1126/science.1149345

[8] Sandin SA, Smith JE, DeMartini EE, Dinsdale EA, Donner SD, Friedlander AM, et al Baselines and degradation of coral reefs in the Northern line islands. PLOS ONE. 2008;3(2):e1548 doi: 10.1371/journal.pone.0001548 18301734 10.1371/journal.pone.0001548 PMC2244711

[9] Friedlander AM, DeMartini EE. Contrasts in density, size, and biomass of reef fishes between the northwestern and the main Hawaiian islands: the effects of fishing down apex predators. Marine Ecology Progress Series. 2002;230:253–64.

[10] Knowlton N, Jackson JBC. Shifting baselines, local impacts, and global change on coral reefs. PLOS Biology. 2008;6(2):e54 doi: 10.1371/journal.pbio.0060054 18303956 10.1371/journal.pbio.0060054 PMC2253644

[11] Friedlander AM, Sandin SA, DeMartini EE, Sala E. Spatial patterns of the structure of reef fish assemblages at a pristine atoll in the central Pacific. Marine Ecology Progress Series. 2010;410:219–31.

[12] Vroom PS, Musburger CA, Cooper SW, Maragos JE, Page-Albins KN, Timmers MAV. Marine biological community baselines in unimpacted tropical ecosystems: spatial and temporal analysis of reefs at Howland and Baker Islands. Biodiversity and Conservation. 2010;19(3):797–812. doi: 10.1007/s10531-009-9735-y

[13] DeMartini EE, Friedlander AM, Sandin SA, Sala E. Differences in fish-assemblage structure between fished and unfished atolls in the northern Line Islands, central Pacific. Marine Ecology Progress Series. 2008;365:199–215.

[14] Chernova NV, Friedlander AM, Turchik A, Sala E. Franz Josef Land: extreme northern outpost for Arctic fishes. PeerJ. 2014;2:e692 doi: 10.7717/peerj.692 25538869 10.7717/peerj.692 PMC4266852

[15] Nonato EF, Brito TAS, De Paiva PC, Petti MAV, Corbisier TN. Benthic megafauna of the nearshore zone of Martel Inlet (King George Island, South Shetland Islands, Antarctica): depth zonation and underwater observations. Polar Biology. 2000;23(8):580–8. doi: 10.1007/s003000000129

[16] Dayton PK, Mordida B, Bacon F. Polar marine communities. American Zoologist. 1994;34(1):90–9.

[17] Brown KM, Fraser KP, Barnes DK, Peck LS. Links between the structure of an Antarctic shallow-water community and ice-scour frequency. Oecologia. 2004;141(1):121–9. doi: 10.1007/s00442-004-1648-6 15338266 10.1007/s00442-004-1648-6

[18] McCauley DJ, Pinsky ML, Palumbi SR, Estes JA, Joyce FH, Warner RR. Marine defaunation: Animal loss in the global ocean. Science. 2015;347(6219). doi: 10.1126/science.1255641 25593191 10.1126/science.1255641

[19] Lewison RL, Crowder LB, Wallace BP, Moore JE, Cox T, Zydelis R, et al Global patterns of marine mammal, seabird, and sea turtle bycatch reveal taxa-specific and cumulative megafauna hotspots. Proceedings of the National Academy of Sciences. 2014;111(14):5271–6. doi: 10.1073/pnas.1318960111 24639512 10.1073/pnas.1318960111 PMC3986184

[20] Glass JP. The fishery and biology of the rock lobster Jasus tristani at the Tristan da Cunha island group [MS Thesis]: Cape Peninsula University of Technology; 2014.

[21] Wanless RM, Scott S, Sauer WHH, Andrew TG, Glass JP, Godfrey B, et al Semi-submersible rigs: a vector transporting entire marine communities around the world. Biological Invasions. 2009;12(8):2573–83. doi: 10.1007/s10530-009-9666-2

[22] Guggenheim DE, Glass T, editors. Disaster at Nightingale–the wreck of the MS Oliva at the world's remotest island: Lessons learned for resource managers in remote areas. International Oil Spill Conference Proceedings; 2014: American Petroleum Institute.

[23] Doolittle DF, Li WK, Wood AM. Wintertime abundance of picoplankton in the Atlantic sector of the Southern Ocean. Nova Hedwigia. 2008;133:147–60.

[24] Deacon GER. Physical and biological zonation in the Southern Ocean. Deep-Sea Research. 1982;29(1A):1–15.

[25] Lutjeharms J, Walters N, Allanson B. Oceanic frontal systems and biological enhancement In: Siegfried W, Condy P, Laws R, editors. Antarctic Nutrient Cycles and Food Webs. Berlin Heidelberg: Springer-Verlag; 1985.

[26] Laubscher RK, Perissinotto R, McQuaid CD. Phytoplankton production and biomass at frontal zones in the Atlantic sector of the Southern Ocean. Polar Biology. 1993;13:471–81.

[27] Bakun A. Fronts and eddies as key structures in the habitat of marine fish larvae: opportunity, adaptive response and competitive advantage. Scientia Marina. 2006;70(S2):105–22.

[28] Block BA, Jonsen ID, Jorgensen SJ, Winship AJ, Shaffer SA, Bograd SJ, et al Tracking apex marine predator movements in a dynamic ocean. Nature. 2011;475(7354):86–90. http://www.nature.com/nature/journal/v475/n7354/abs/nature10082-f1.2.html—supplementary-information. doi: 10.1038/nature10082 21697831 10.1038/nature10082

[29] Bost CA, Cotté C, Bailleul F, Cherel Y, Charrassin JB, Guinet C, et al The importance of oceanographic fronts to marine birds and mammals of the southern oceans. Journal of Marine Systems. 2009;78(3):363–76. doi: 10.1016/j.jmarsys.2008.11.022

[30] Etnoyer P, Canny D, Mate B, Morgan L. Persistent pelagic habitats in the Baja California to Bering Sea (B2B) ecoregion. Oceanography 2004;17(1):90–101. http://dx.doi.org/10.5670/oceanog.2004.71.

[31] Weng KC, Foley DG, Ganong JE, Perle C, Shillinger GL, Block BA. Migration of an upper trophic level predator, the salmon shark *Lamna ditropis*, between distant ecoregions. Marine Ecology Progress Series. 2008;372:253–64.

[32] Raymond B, Shaffer SA, Sokolov S, Woehler EJ, Costa DP, Einoder L, et al Shearwater foraging in the southern ocean: The roles of prey availability and winds. PLOS ONE. 2010;5(6):e10960 doi: 10.1371/journal.pone.0010960 20532034 10.1371/journal.pone.0010960 PMC2881033

[33] Robinson PW, Costa DP, Crocker DE, Gallo-Reynoso JP, Champagne CD, Fowler MA, et al Foraging behavior and success of a mesopelagic predator in the northeast Pacific Ocean: insights from a data-rich species, the northern elephant seal. PloS one. 2012;7(5):e36728 doi: 10.1371/journal.pone.0036728 22615801 10.1371/journal.pone.0036728 PMC3352920

[34] Queiroz N, Humphries NE, Mucientes G, Hammerschlag N, Lima FP, Scales KL, et al Ocean-wide tracking of pelagic sharks reveals extent of overlap with longline fishing hotspots. Proc Natl Acad Sci U S A. 2016;113(6):1582–7. doi: 10.1073/pnas.1510090113 . 26811467 10.1073/pnas.1510090113 PMC4760806

[35] Polovina JJ, Howell E, Kobayashi DR, Seki MP. The transition zone chlorophyll front, a dynamic global feature defining migration and forage habitat for marine resources. Progress in Oceanography. 2001;49(1–4):469–83. https://doi.org/10.1016/S0079-6611(01)00036-2.

[36] Zainuddin M, Kiyofuji H, Saitoh K, Saitoh S-I. Using multi-sensor satellite remote sensing and catch data to detect ocean hot spots for albacore (*Thunnus alalunga*) in the northwestern North Pacific. Deep Sea Research Part II: Topical Studies in Oceanography. 2006;53(3–4):419–31. https://doi.org/10.1016/j.dsr2.2006.01.007.

[37] Scales KL, Miller PI, Hawkes LA, Ingram SN, Sims DW, Votier SC. On the Front Line: frontal zones as priority at‐sea conservation areas for mobile marine vertebrates. Journal of Applied Ecology. 2014;51(6):1575–83.

[38] Andrew T, Hecht T, Heemstra P, Lutjeharms J. Fishes of the Tristan da Cunha group and Gough Island, South Atlantic Ocean. Ichthyological Bulletin of the JLB Smith Institute of Ichthyology. 1995;63:1–43.

[39] Scott S. A Biophysical Profile of the Tristan da Cunha Archipelago. A report to The Pew Charitable Trusts. 2016.

[40] Hamilton SL, Caselle JE, Malone DP, Carr MH. Incorporating biogeography into evaluations of the Channel Islands marine reserve network. Proceedings of the National Academy of Sciences. 2010;107(43):18272–7. 10.1073/pnas.0908091107 PMC2973008 20176956

[41] Letessier TB, Meeuwig JJ, Gollock M, Groves L, Bouchet PJ, Chapuis L, et al Assessing pelagic fish populations: The application of demersal video techniques to the mid-water environment. Methods in Oceanography. 2013;8:41–55. http://dx.doi.org/10.1016/j.mio.2013.11.003.

[42] Willis TJ, Babcock RC. A baited underwater video system for the determination of relative density of carnivorous reef fish. Marine and Freshwater Research. 2000;51(8):755–63. https://doi.org/10.1071/MF00010.

[43] Froese R, Pauly D. FishBase. 2011. World Wide Web electronic publication Available at: http://www/fishbase.org (accessed 22 February 2011). 2011.

[44] Pollock DE. Spiny lobsters at Tristan da Cunha, South Atlantic: inter-island variations in growth and population structure. South African Journal of Marine Science. 1991;10(1):1–12. doi: 10.2989/02577619109504614

[45] Friedlander AM, Ballesteros E, Caselle JE, Gaymer CF, Palma AT, Petit I, et al Marine biodiversity in Juan Fernández and Desventuradas Islands, Chile: global endemism hotspots. PLOS ONE. 2016;11(1):e0145059 doi: 10.1371/journal.pone.0145059 26734732 10.1371/journal.pone.0145059 PMC4703205

[46] Caselle JE, Rassweiler A, Hamilton SL, Warner RR. Recovery trajectories of kelp forest animals are rapid yet spatially variable across a network of temperate marine protected areas. Sci Rep. 2015;5:14102 doi: 10.1038/srep14102 ; PubMed Central PMCID: PMCPMC4642697. 26373803 10.1038/srep14102 PMC4642697

[47] Friedlander AM, Ballesteros E, Beets J, Berkenpas E, Gaymer CF, Gorny M, et al Effects of isolation and fishing on the marine ecosystems of Easter Island and Salas y Gómez, Chile. Aquatic Conservation: Marine and Freshwater Ecosystems. 2013;23(4):515–31. doi: 10.1002/aqc.2333

[48] Aburto-Oropeza O, Erisman B, Galland G, Mascareñas-Osorio I, Sala E, Ezcurra E. Large recovery of fish biomass in a no-take marine reserve. Plos ONE. 2011;6(8):e23601 doi: 10.1371/journal.pone.0023601 21858183 10.1371/journal.pone.0023601 PMC3155316

[49] Babcock RC, Phillips JC, Lourey M, Clapin G. Increased density, biomass and egg production in an unfished population of Western Rock Lobster (Panulirus cygnus) at Rottnest Island, Western Australia. Marine and Freshwater Research. 2007;58(3):286–92. https://doi.org/10.1071/MF06204.

[50] Freeman DJ, Macdiarmid AB, Taylor RB, Davidson RJ, Grace RV, Haggitt TR, et al Trajectories of spiny lobster *Jasus edwardsii* recovery in New Zealand marine reserves: is settlement a driver? Environmental Conservation. 2012;39(03):295–304.

[51] Reed DC, Rassweiler A, Arkema KK. Biomass rather than growth rate determines variation in net primary production by giant kelp. Ecology. 2008;89(9):2493–505. doi: 10.1890/07-1106.1 18831171 10.1890/07-1106.1

[52] Dayton PK, Tegner MJ, Edwards PB, Riser KL. Sliding baselines, ghosts, and reduced expectations in kelp forest communities. Ecological Applications. 1998;8(2):309–22. doi: 10.1890/1051-0761(1998)008[0309:SBGARE]2.0.CO;2

[53] Van Tussenbroek BI. Plant and frond dynamics of the giant kelp, *Macrocystis pyrifera*, forming a fringing zone in the Falkland Islands. European Journal of Phycology. 1993;28(3):161–5. doi: 10.1080/09670269300650251

[54] Reed DC, Rassweiler AR, Arkema K. Density derived estimates of standing crop and net primary production in the giant kelp *Macrocystis pyrifera*. Marine Biology. 2009;156:2077–83. doi: 10.1007/s00227-009-1238-6 24391235 10.1007/s00227-009-1238-6 PMC3873067

[55] Jackson GA. Modelling the growth and harvest yield of the giant kelp *Macrocystis pyrifera*. Marine Biology. 1987;95:611–24.

[56] Tegner MJ, Dayton PK, Edwards PB, Riser KL. Large-scale, low-frequency oceanographic effects on kelp forest succession: a tale of two cohorts. Marine Ecology Progress Series. 1997;146:117–34.

[57] Rodriguez GE, Rassweiler A, Reed DC, Holbrook SJ. The importance of progressive senescence in the biomass dynamics of giant kelp (*Macrocystis pyrifera*). Ecology. 2013;94(8):1848–58. 24015528 10.1890/12-1340.1

[58] Utter BD, Denny MW. Wave-induced forces on the giant kelp *Macrocystis pyrifera* (Agardh): Field test of a computational model. The Journal of Experimental Biology. 1996;199:2645–54. 9320580 10.1242/jeb.199.12.2645

[59] Dayton PK, Tegner MJ. Catastrophic storms, El Niño, and patch stability in a southern California kelp community. Science. 1984;224(4646):283–5. doi: 10.1126/science.224.4646.283 17734914 10.1126/science.224.4646.283

[60] Seymour R, Tegner M, Dayton P, Parnell P. Storm wave induced mortality of giant kelp, *Macrocystis pyrifera*, in southern California. Estuarine, Coastal and Shelf Science. 1989;28(3):277–92.

[61] Orsi AH, Whitworth III, Nowlin WD Jr.. On the meridional extent and fronts of the Antarctic Circumpolar Current. Deep Sea Research I. 1995;42:641–73.

[62] Deacon G. A general account of the hydrology of the North Atlantic ocean. Discovery Rep. 1933;7.

[63] Jackson GA. Nutrients and production of giant kelp, *Macrocystis pyrifera*, off southern California1. Oceanography. 1977;22(6).

[64] Bell TW, Cavanaugh KC, Reed DC, Siegel DA. Geographical variability in the controls of giant kelp biomass dynamics. Journal of Biogeography. 2015;42(10):2010–21.

[65] Reed DC, Rassweiler A, Carr MH, Cavanaugh KC, Malone DP, Siegel DA. Wave disturbance overwhelms top‐down and bottom‐up control of primary production in California kelp forests. Ecology. 2011;92(11):2108–16. 22164835 10.1890/11-0377.1

[66] Heydorn AEF. The South Atlantic rock lobster *Jasus tristani* at Vema Seamount, Gough Island and Tristan da Cunha: Division of Sea Fisheries; 1969.

[67] Andrew T. Feeding biology of *Acantholatris monodactylus* (Pisces: Cheilodactylidae) at Tristan da Cunha and Gough Island, South Atlantic. S Afr J Antarct Res. 1992;22:41–9.

[68] Barrientos CA, Gonzalez MT, Moreno CA. Geographical differences in the feeding patterns of red rockfish (*Sebastes capensis*) along South American coasts. Fishery Bulletin. 2006;104(4):489–97.

[69] Dubiaski-Silva J, Masunari S. Ontogenetic and seasonal variation in the diet of marimbá, *Diplodus argenteus* (Valenciennes, 1830)(Pisces, Sparidae) associated with the beds of *Sargassum cymosum* C. Agardh, 1820 (Phaeophyta) at Ponta das Garoupas, Bombinhas, Santa Catarina. Journal of Coastal Research. 2006:1190–2.

[70] Baxter JL. A study of the yellowtail *Seriola dorsalis* (Gill). State of California Department of Fish and Game—Fish Bulletin. 1960;110:1–96.

[71] Ebert DA. Diet of the seven gill shark *Notorynchus cepedianus* in the temperate coastal waters of southern Africa. South African Journal of Marine Science. 1991;11(1):565–72.

[72] Smith C. Diet of *Octopus vulgaris* in False Bay, South Africa. Marine Biology. 2003;143(6):1127–33.

[73] Borrvall C, Ebenman B, Tomas Jonsson TJ. Biodiversity lessens the risk of cascading extinction in model food webs. Ecology Letters. 2000;3(2):131–6. doi: 10.1046/j.1461-0248.2000.00130.x

[74] Eklöf A, Ebenman BO. Species loss and secondary extinctions in simple and complex model communities. Journal of Animal Ecology. 2006;75(1):239–46. doi: 10.1111/j.1365-2656.2006.01041.x 16903061

[75] Johnson CR, Banks SC, Barrett NS, Cazassus F, Dunstan PK, Edgar GJ, et al Climate change cascades: Shifts in oceanography, species' ranges and subtidal marine community dynamics in eastern Tasmania. Journal of Experimental Marine Biology and Ecology. 2011;400(1–2):17–32. https://doi.org/10.1016/j.jembe.2011.02.032.

[76] Wernberg T, Russell BD, Moore PJ, Ling SD, Smale DA, Campbell A, et al Impacts of climate change in a global hotspot for temperate marine biodiversity and ocean warming. Journal of Experimental Marine Biology and Ecology. 2011;400(1–2):7–16. https://doi.org/10.1016/j.jembe.2011.02.021.

[77] Krumhansl KA, Okamoto DK, Rassweiler A, Novak M, Bolton JJ, Cavanaugh KC, et al Global patterns of kelp forest change over the past half-century. Proceedings of the National Academy of Sciences. 2016;113(48):13785–90. 10.1073/pnas.1606102113 PMC5137772 27849580

[78] Reed D, Washburn L, Rassweiler A, Miller R, Bell T, Harrer S. Extreme warming challenges sentinel status of kelp forests as indicators of climate change. Nature Communications. 2016;7. 10.1038/ncomms13757 PMC5159872 27958273

[79] Breen PA, Mann KH. Changing lobster abundance and the destruction of kelp beds by sea urchins. Marine Biology. 1976;34(2):137–42. doi: 10.1007/bf00390755

[80] Shears NT, Babcock RC. Marine reserves demonstrate top-down control of community structure on temperate reefs. Oecologia. 2002;132(1):131–42. doi: 10.1007/s00442-002-0920-x 28547276 10.1007/s00442-002-0920-x

[81] Lafferty KD. Fishing for lobsters indirectly increases epidemics in sea urchins. Ecological Applications. 2004;14(5):1566–73.

[82] Ling SD, Johnson CR, Frusher SD, Ridgway KR. Overfishing reduces resilience of kelp beds to climate-driven catastrophic phase shift. Proceedings of the National Academy of Sciences. 2009;106(52):22341–5. doi: 10.1073/pnas.0907529106 20018706 10.1073/pnas.0907529106 PMC2793314

[83] Jordán F, Scheuring I, Vida G. Species positions and extinction dynamics in simple food webs. Journal of Theoretical Biology. 2002;215(4):441–8. doi: 10.1006/jtbi.2001.2523 12069488 10.1006/jtbi.2001.2523

[84] Dunne JA, Williams RJ, Martinez ND. Network structure and biodiversity loss in food webs: robustness increases with connectance. Ecology Letters. 2002;5(4):558–67. doi: 10.1046/j.1461-0248.2002.00354.x

[85] Worm B, Lotze HK, Myers RA. Predator diversity hotspots in the blue ocean. Proc Natl Acad Sci USA 2003;100(17):9884–8. doi: 10.1073/pnas.1333941100 12907699 10.1073/pnas.1333941100 PMC187874

[86] Bradshaw CJA, Hindell MA, Sumner MD, Michael KJ. Loyalty pays: potential life history consequences of fidelity to marine foraging regions by southern elephant seals. Animal Behaviour. 2004;68(6):1349–60. https://doi.org/10.1016/j.anbehav.2003.12.013.

[87] Friedlander AM, Caselle JE, Ballesteros E, Brown EK, Turchik A, Sala E. The Real Bounty: Marine Biodiversity in the Pitcairn Islands. PLOS ONE. 2014;9(6):e100142 doi: 10.1371/journal.pone.0100142 24963808 10.1371/journal.pone.0100142 PMC4070931

[88] Anderson ORJ, Small CJ, Croxall JP, Dunn EK, Sullivan BJ, Yates O, et al Global seabird bycatch in longline fisheries. Endangered Species Research. 2011;14(2):91–106.

