# Supplementary material for: First quantification of subtidal community structure at Tristan da Cunha Islands in the remote South Atlantic: from kelp forests to the deep sea
Source: PLoS One. 2018 Mar 29;13(3):e0195167. doi: 10.1371/journal.pone.0195167 (PMC5875861; doi:10.1371/journal.pone.0195167)

S1 Fig. Size structure from SCUBA surveys. Spatial variation in size structure of lobsters, giant kelp, and two common nearshore fish species estimated using visual SCUBA surveys. Shown are size frequency histograms for each species pooled at the island-level. Arrows above the histogram depict the estimated mean size at each island. Values on plots are the mean size ± 1 standard error of the mean.

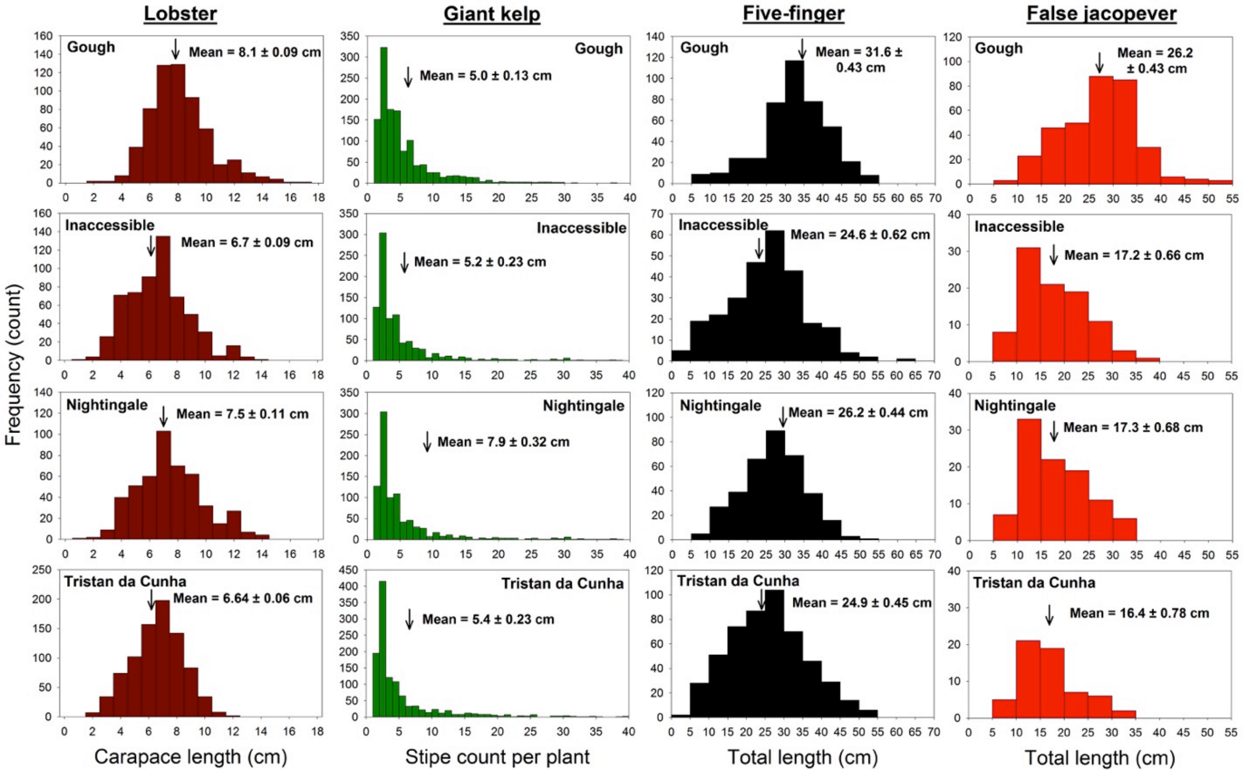

Supplement: S1 Fig — Spatial variation in size structure of lobsters, giant kelp, and two common nearshore fish species estimated using visual SCUBA surveys. Shown are size frequency histograms for each species pooled at the island-level. Arrows above the histogram depict the estimated mean size at each island. Values on plots are the mean size ± 1 standard error of the mean. (PDF) [file pone.0195167.s001.pdf]
