# Supplementary material for: First quantification of subtidal community structure at Tristan da Cunha Islands in the remote South Atlantic: from kelp forests to the deep sea
Source: PLoS One. 2018 Mar 29;13(3):e0195167. doi: 10.1371/journal.pone.0195167 (PMC5875861; doi:10.1371/journal.pone.0195167)

S2 Fig. Substrate and habitat relief. Substrate composition and habitat complexity estimated using visual SCUBA surveys in nearshore kelp forests (10 and 20 m depth) in the Tristan da Cunha Islands. Shown are the percent cover of (A) four different substrate categories and (B) vertical relief in four different relief categories.

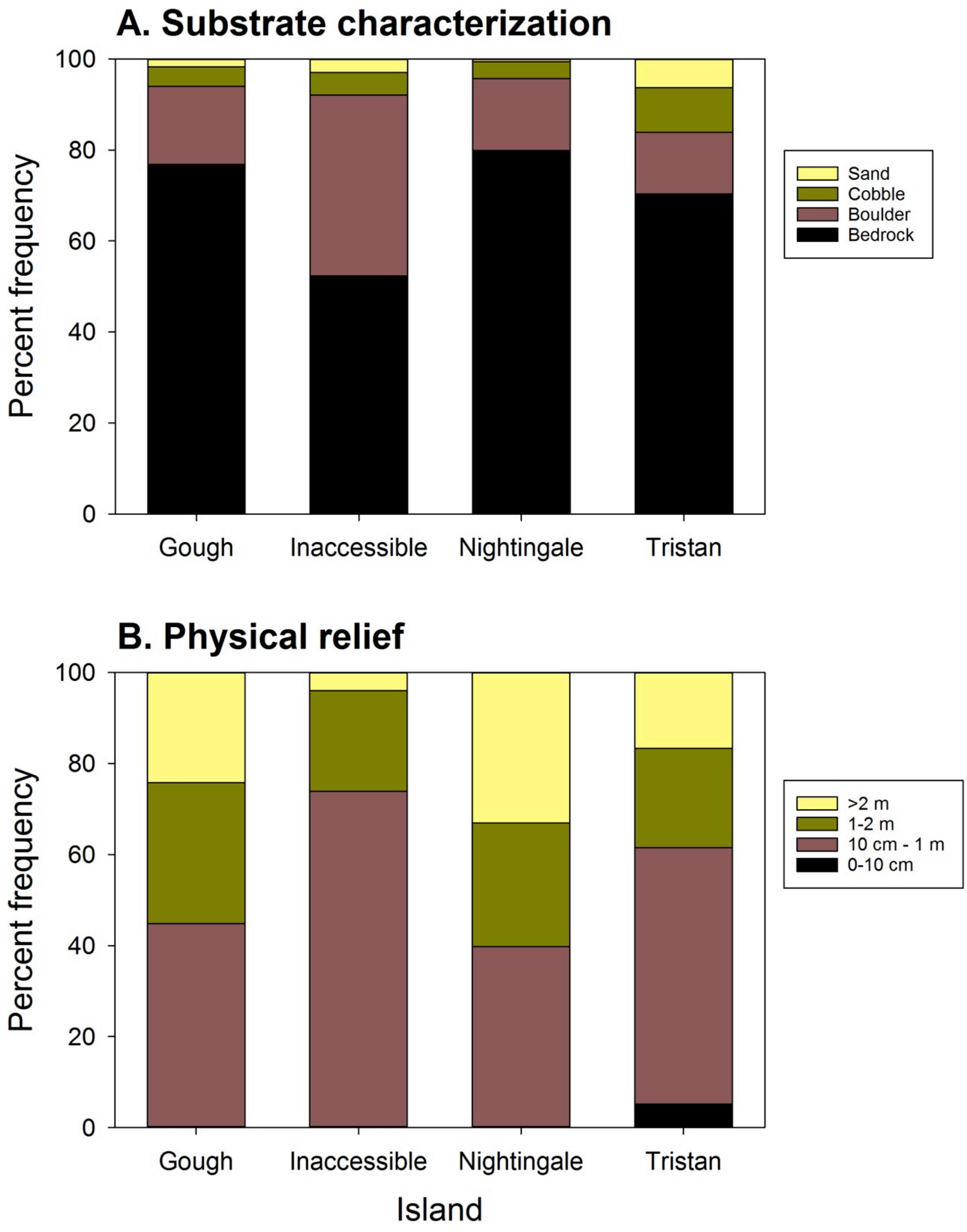

Supplement: S2 Fig — Substrate composition and habitat complexity estimated using visual SCUBA surveys in nearshore kelp forests (10 and 20 m depth) in the Tristan da Cunha Islands. Shown are the percent cover of (A) four different substrate categories and (B) vertical relief in four different relief categories. (PDF) [file pone.0195167.s002.pdf]
