# Supplementary material for: First quantification of subtidal community structure at Tristan da Cunha Islands in the remote South Atlantic: from kelp forests to the deep sea
Source: PLoS One. 2018 Mar 29;13(3):e0195167. doi: 10.1371/journal.pone.0195167 (PMC5875861; doi:10.1371/journal.pone.0195167)

S3 Fig. Pelagic BRUV species. Photos depicting representative species observed on pelagic camera drops at the Tristan da Cunha Islands. (A) Krill school (*Euphausids spp.*), Gough; (B) Blue sharks (*Prionace glauca.*), Nightingale; (C) Recently born blue shark (*Prionace glauca.*), Tristan da Cunha; (D) Porbeagle shark (*Lamnin nasus*), Tristan da Cunha; (E) Southern horse mackerel (*Trachurus longimanus*), Inaccessible; (F) Yellowtail amberjack school (*Seriola lalandi*), Tristan da Cunha; (G) Striped marlin (*Kajikia albida*), Tristan da Cunha; (H) Yellowfin tuna (*Thunnus albacares*), Tristan da Cunha; (I) Loggerhead turtle (*Caretta caretta.*), Tristan da Cunha; (J) Subantarctic fur seal (*Arctocephalus tropicalis*), Gough; (K) Dusky dolphin (*Lagenorhynchus obscurus*), Gough; (L) Shepherd's beaked whale (*Tasmacetus shepherdi*); Inaccessible.

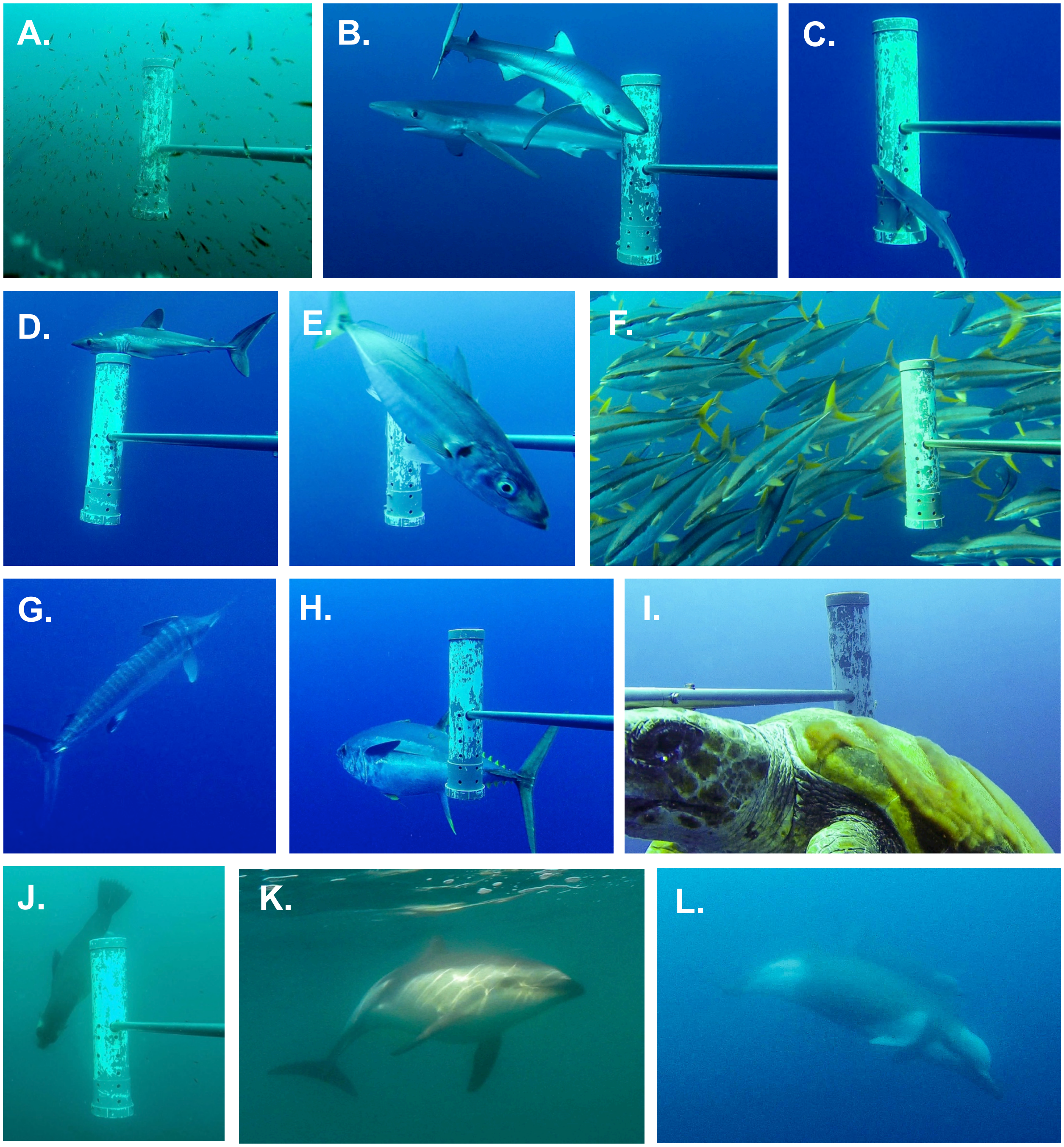

Supplement: S3 Fig — Photos depicting representative species observed on pelagic camera drops at the Tristan da Cunha Islands. (A) Krill school (Euphausids spp.), Gough; (B) Blue sharks (Prionace glauca.), Nightingale; (C) Recently born blue shark (Prionace glauca.), Tristan da Cunha; (D) Porbeagle shark (Lamnin nasus), Tristan da Cunha; (E) Southern horse mackerel (Trachurus longimanus), Inaccessible; (F) Yellowtail amberjack school (Seriola lalandi), Tristan da Cunha; (G) Striped marlin (Kajikia albida), Tristan da Cunha; (H) Yellowfin tuna (Thunnus albacares), Tristan da Cunha; (I) Loggerhead turtle (Caretta caretta.), Tristan da Cunha; (J) Subantarctic fur seal (Arctocephalus tropicalis), Gough; (K) Dusky dolphin (Lagenorhynchus obscurus), Gough; (L) Shepherd’s beaked whale (Tasmacetus shepherdi); Inaccessible. (PDF) [file pone.0195167.s003.pdf]
