# Supplementary material for: First quantification of subtidal community structure at Tristan da Cunha Islands in the remote South Atlantic: from kelp forests to the deep sea
Source: PLoS One. 2018 Mar 29;13(3):e0195167. doi: 10.1371/journal.pone.0195167 (PMC5875861; doi:10.1371/journal.pone.0195167)

S4 Fig. Deep sea species. Photos depicting representative fish and habitat-forming benthic species observed on deep-sea camera drops at the Tristan da Cunha Islands. (A) Bluntnose sixgill shark (*Hexanchus griseus*), (B) Lantern shark (*Etmopterus* sp., likely *granulosus*), (C) Cutthroat eel (*Synaphobranchus* sp., likely *brevidorsalis*), (D) Deepwater cod (*Physiculus karrerae*), (E) Roughy (*Beryx decadactylus*), (F) Oreo dory (*Neocyttus* sp.), (G) Soldier (*Heliocolenus mouchezi*) and Octopus (*Octopus vulgaris*), (H) Southern butterfish (*Hyperoglyphe antarctica.*), (I) Oval driftfish (*Schedophilus velaini*), (J) Whip coral, (K) Deepwater gorgonian, (L) Field of gorgonians and crinoids, (M) Sea pens.

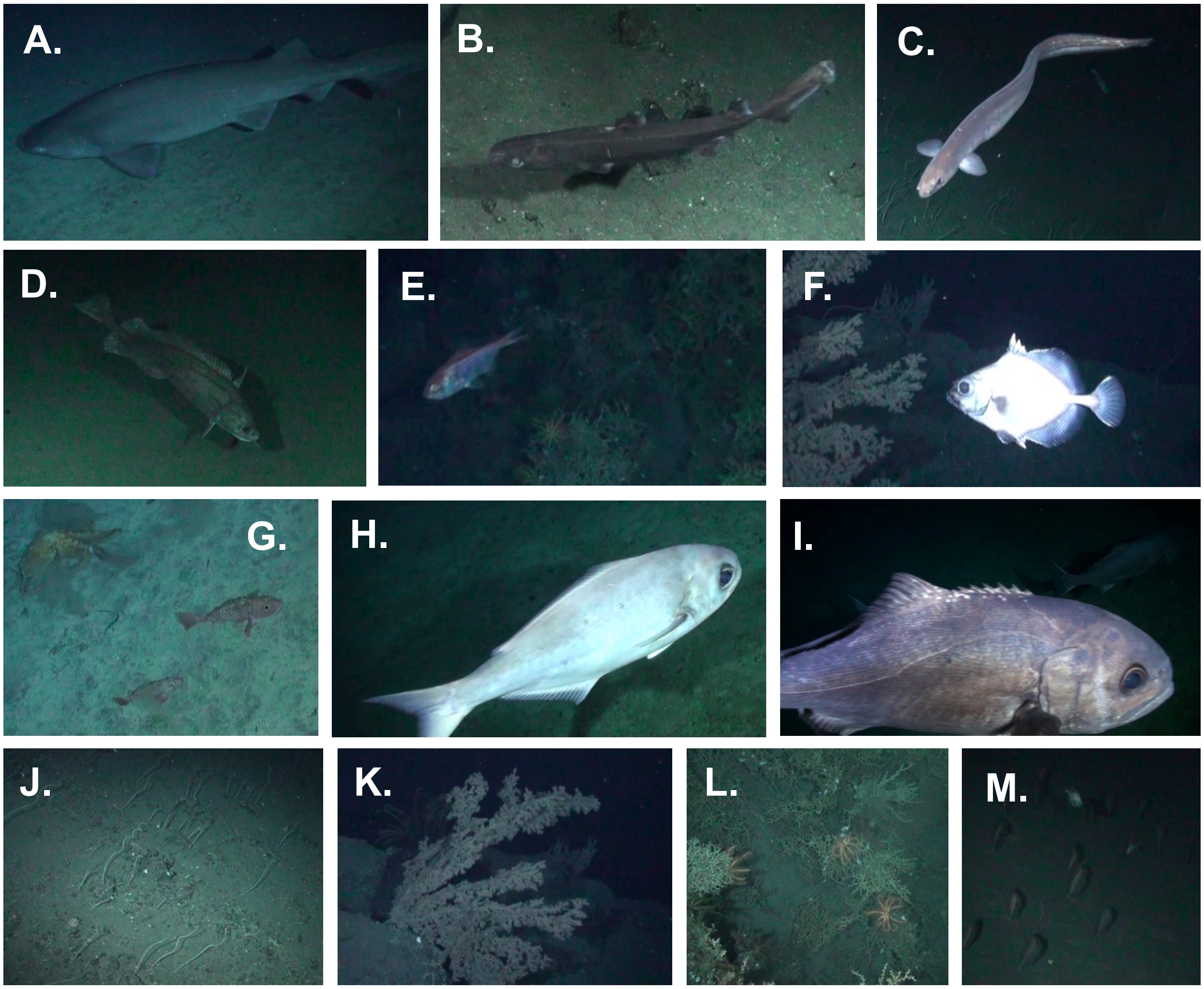

Supplement: S4 Fig — Photos depicting representative fish and habitat-forming benthic species observed on deep-sea camera drops at the Tristan da Cunha Islands. (A) Bluntnose sixgill shark (Hexanchus griseus), (B) Lantern shark (Etmopterus sp., likely granulosus), (C) Cutthroat eel (Synaphobranchus sp., likely brevidorsalis), (D) Deepwater cod (Physiculus karrerae), (E) Roughy (Beryx decadactylus), (F) Oreo dory (Neocyttus sp.), (G) Soldier (Heliocolenus mouchezi) and Octopus (Octopus vulgaris), (H) Southern butterfish (Hyperoglyphe antarctica.), (I) Oval driftfish (Schedophilus velaini), (J) Whip coral, (K) Deepwater gorgonian, (L) Field of gorgonians and crinoids, (M) Sea pens. (PDF) [file pone.0195167.s004.pdf]
