# Supplementary material for: First quantification of subtidal community structure at Tristan da Cunha Islands in the remote South Atlantic: from kelp forests to the deep sea
Source: PLoS One. 2018 Mar 29;13(3):e0195167. doi: 10.1371/journal.pone.0195167 (PMC5875861; doi:10.1371/journal.pone.0195167)
Supplement: S1 Table — Details of the deep-sea drop camera deployments for all stations surveyed in the Tristan da Cunha Islands group. (PDF) [file pone.0195167.s005.pdf]

S1 Table. Deep-sea drop camera deloyments. Details of the deep-sea drop camera deployments for all stations surveyed in the Tristan da Cunha Islands group.

Drop No.	Date	Island	Latitude	Longitude	Depth (m)	Time	Duration (min)
T1	01/16/17	Tristan	-37.03262	-12.31115	893	14:55	120
T2	01/17/17	Tristan	-37.02890	-12.26669	1173	07:21	120
T3	01/17/17	Tristan	-37.03608	-12.30570	656	10:18	120
T4	01/18/17	Tristan	-37.12096	-12.39176	1075	09:55	120
T5	01/18/17	Tristan	-37.14068	-12.38775	953	11:53	120
T6	01/21/17	Gough	-40.25043	-9.88091	1414	08:24	120
T7	01/21/17	Gough	-40.27888	-9.85603	1404	12:55	120
T8	01/22/17	Gough	-40.34076	-9.85285	714	08:49	120
T9	01/22/17	Gough	-40.36598	-9.85951	467	10:56	60
T10	01/23/17	Gough	-40.25772	-9.90926	190	13:15	120
T11	01/23/17	Gough	-40.26271	-9.88240	1027	13:29	120
T12	01/26/17	Tristan	-37.19624	-12.24193	1027	11:04	120
T13	01/26/17	Tristan	-37.17244	-12.22194	1122	14:13	120
T14	01/28/17	Inaccessible	-37.27441	-12.73408	225	08:39	120
T15	01/28/17	Inaccessible	-37.26014	-12.71080	164	10:42	120
T16	01/29/17	Nightingale	-37.39484	-12.55375	1227	07:26	120
T17	01/29/17	Nightingale	-37.43821	-12.53270	608	10:14	60
T18	01/30/17	Nightingale	-37.44452	-12.44944	418	07:10	120
T19	01/30/17	Nightingale	-37.40688	-12.43674	708	10:24	120
T20	01/31/17	Tristan	-37.19418	-12.31482	994	08:00	120
T21	01/31/17	Tristan	-37.16568	-12.36713	712	10:50	45
T22	02/01/17	Tristan	-37.11137	-12.17371	1200	07:21	120
T23	02/01/17	Tristan	-37.09369	-12.18232	1203	10:48	120
