# Supplementary material for: First quantification of subtidal community structure at Tristan da Cunha Islands in the remote South Atlantic: from kelp forests to the deep sea
Source: PLoS One. 2018 Mar 29;13(3):e0195167. doi: 10.1371/journal.pone.0195167 (PMC5875861; doi:10.1371/journal.pone.0195167)
Supplement: S2 Table — Biomass (tonnes ha-1) and density (no. 100 m2) of fish, conspicuous benthic invertebrates, and kelps observed on SCUBA surveys at 10 and 20 m depth in the Tristan da Cunha Islands. Values are means ± 1 standard error of the mean for each island. (PDF) [file pone.0195167.s006.pdf]

S2 Table. Biomass and density from SCUBA surveys. Biomass (tonnes ha^{-1}) and density (no. 100 m^2) of fish, conspicuous benthic invertebrates, and kelps observed on SCUBA surveys at 10 and 20 m depth in the Tristan da Cunha Islands. Values are means ± 1 standard error of the mean for each island

Species	Gough	Inaccessible	Nightingale	Tristan
**A. Fish Biomass**				
Total fish biomass	1.95 ± 0.18	1.50 ± 0.27	2.75 ± 1.05	1.46 ± 0.29
Five-finger (*Nemadactylus monodactylus*)	1.1 ± 0.10	0.56 ± 0.18	0.99 ± 0.32	0.69 ± 0.12
Tristan Wrasse (*Suezichthys ornatus*)	0.02 ± 0.008	0.33 ± 0.05	0.15 ± 0.07	0.21 ± 0.04
False Jacopever (*Sebastes capensis*)	0.55 ± 0.05	0.063 ± 0.02	0.071 ± 0.02	0.024 ± 0.005
Telescopefish (*Mendosoma lineatum*)	0.30 ± 0.07	0.06 ± 0.03	0.018 ± 0.01	0.005 ± 0.002
Klipfish (*Bovicthus diacanthus*)	0.018 ± 0.003	0.0001 ± 1e-4	0.0003 ± 2e-4	0 ± 0
Soldier (*Helicolenus mouchezi*)	0.002 ± 0.002	0.01 ± 0.01	0 ± 0	0 ± 0
Invasive porgy (*Diplodus argenteus*)	0 ± 0	0.009 ± 0.006	0 ± 0	0.036 ± 0.01
Yellowtail (*Seriola lalandi*)	0 ± 0	0.46 ± 0.24	1.52 ± 0.74	0.51 ± 0.28
**B. Fish Density**				
Total fish density	164.5 ± 16.6	201. 1 ± 15.9	124. 4 ± 36.4	138.0 ± 21.0
Five-finger (*Nemadactylus monodactylus*)	39.1 ± 4.0	42.0 ± 12.4	65.6 ± 18.2	52.0 ± 7.6
Tristan Wrasse (*Suezichthys ornatus*)	1.5 ± 0.4	146.1 ± 25.4	49.4 ± 19.7	75.4 ± 16.3
False Jacopever (*Sebastes capensis*)	18.9 ± 1.9	6.3 ± 1.3	7.0 ± 1.3	2.6 ± 0.5
Telescopefish (*Mendosoma lineatum*)	101.1 ± 16.0	4.7 ± 2.9	1.1 ± 0.7	0.4 ± 0.2
Klipfish (*Bovicthus diacanthus*)	3.9 ± 0.5	0.1 ± 0.1	0.1 ± 0.08	0 ± 0
Soldier (*Helicolenus mouchezi*)	0.04 ± 0.04	0.12 ± 0.12	0 ± 0	0 ± 0
Invasive porgy (*Diplodus argenteus*)	0 ± 0	1.3 ± 1.0	0 ± 0	7.1 ± 3.5
Yellowtail (*Seriola lalandi*)	0 ± 0	0.5 ± 0.2	1.2 ± 0.6	0.5 ± 0.2
**C. Benthic species density and biomass**				
Giant kelp (*Macrocystis pyrifera*)	53.9 ± 10.1	53.8 ± 12.6	44.7 ± 15.6	49.1 ± 14.4
Giant kelp stipes	265.6 ± 29.8	279.5 ± 67.2	354.3 ± 117.5	262.9 ± 64.5
Pale kelp (*Laminaria pallida*)	235.9 ± 24.1	388.8 ± 56.1	511.8 ± 147.0	533.6 ± 109.4
Lobster biomass (*Jasus tristani*)	0.15 ± 0.022	0.11 ± 0.019	0.13 ± 0.014	0.085 ± 0.012
Lobster density	6.4 ± 0.8	8.6 ± 1.2	7.2 ± 1.3	8.8 ± 1.3
Sea urchin (*Arbacia dufresnii*)	389.4 ± 62.7	119.5 ± 25.4	60.1 ± 25.2	23.2 ± 7.2
Pink urchin (*Pseudechinus magellanicus*)	8.0 ± 4.1	0 ± 0	0 ± 0	0 ± 0
Sea star (*Henricia simplex*)	8.2 ± 1.3	6.1 ± 0.9	7.7 ± 4.5	12.6 ± 1.9
Bat star (*Odontaster penicillatus*)	0.54 ± 0.2	0 ± 0	0 ± 0	0 ± 0
Common octopus (*Octopus vulgaris*)	0.25 ± 0.09	0.47 ± 0.2	0.41 ± 0.2	0.13 ± 0.06
Whelk (*Argrobuccinium tristanensis*)	2.5 ± 0.7	0.3 ± 0.2	0.3 ± 0.1	0.54 ± 0.3
Barnacle (*Austromegabalanus sp.*)	0.41 ± 0.42	0.12 ± 0.11	12.5 ± 12.4	0 ± 0
