# Supplementary material for: First quantification of subtidal community structure at Tristan da Cunha Islands in the remote South Atlantic: from kelp forests to the deep sea
Source: PLoS One. 2018 Mar 29;13(3):e0195167. doi: 10.1371/journal.pone.0195167 (PMC5875861; doi:10.1371/journal.pone.0195167)
Supplement: S3 Table — Shown are the top three species contributing to community dissimilarity on each island and the percent contribution of each species to community dissimilarity in each pair-wise comparison. Mean densities of these and other species by site are shown in S2 Table. (PDF) [file pone.0195167.s007.pdf]

S3 Table. Similarity Percentage Analysis (SIMPER) showing the average percent dissimilarity in the fish assemblage structure among the four Tristan da Cunha islands. Shown are the top three species contributing to community dissimilarity on each island and the percent contribution of each species to community dissimilarity in each pair-wise comparison. Mean densities of these and other species by site are shown in Table S2.

**A. Gough vs. Inaccessible – Average dissimilarity = 56.1%**		
**Species**	**Average dissimilarity**	**% Contribution**
Tristan wrasse	23.41	41.7
Telescopefish	17.63	31.4
Five-finger	4.34	7.72
**B. Gough vs. Nightingale – Average dissimilarity = 51.4%**		
**Species**	**Average dissimilarity**	**% Contribution**
Telescopefish	22.47	43.65
Tristan wrasse	12.80	24.86
Five-finger	5.60	10.88
**C. Gough vs. Tristan – Average dissimilarity = 58.99%**		
**Species**	**Average dissimilarity**	**% Contribution**
Telescopefish	4.67	36.75
Tristan wrasse	3.30	28.49
False jacopever	3.14	11.03
**D. Inaccessible vs. Nightingale – Average dissimilarity = 31.67%**		
**Species**	**Average dissimilarity**	**% Contribution**
Tristan wrasse	14.77	46.64
Five-finger	7.13	22.52
Telescopefish	3.57	11.28
**E. Inaccessible vs. Tristan – Average dissimilarity = 27.45%**		
**Species**	**Average dissimilarity**	**% Contribution**
Tristan wrasse	10.46	38.12
Five-finger	5.51	20.07
Invasive porgy	4.08	14.87
**F. Nightingale vs. Tristan – Average dissimilarity = 27.23%**		
**Species**	**Average dissimilarity**	**% Contribution**
Tristan wrasse	8.26	30.34
Five-finger	6.43	23.61
Invasive porgy	5.16	18.97
