# Supplementary material for: First quantification of subtidal community structure at Tristan da Cunha Islands in the remote South Atlantic: from kelp forests to the deep sea
Source: PLoS One. 2018 Mar 29;13(3):e0195167. doi: 10.1371/journal.pone.0195167 (PMC5875861; doi:10.1371/journal.pone.0195167)
Supplement: S4 Table — Shown are the top three species contributing to community dissimilarity on each island and the percent contribution of each species to community dissimilarity in each pair-wise comparison. Mean densities of these and other species by site are shown in S2 Table. (PDF) [file pone.0195167.s008.pdf]

S4 Table. Similarity Percentage Analysis (SIMPER) showing the average percent dissimilarity in the benthic community structure among the four Tristan da Cunha islands. Shown are the top three species contributing to community dissimilarity on each island and the percent contribution of each species to community dissimilarity in each pair-wise comparison. Mean densities of these and other species by site are shown in Table S2.

**A. Gough vs. Inaccessible – Average dissimilarity = 21.1%**		
**Species**	**Average dissimilarity**	**% Contribution**
Sea urchin	7.5	35.7
Giant kelp stipes	4.0	19.0
Pale kelp	3.9	18.9
**B. Gough vs. Nightingale – Average dissimilarity = 32.7%**		
**Species**	**Average dissimilarity**	**% Contribution**
Sea urchin	10.7	32.7
Giant kelp stipes	7.6	23.3
Pale kelp	7.0	21.5
**C. Gough vs. Tristan – Average dissimilarity = 31.5%**		
**Species**	**Average dissimilarity**	**% Contribution**
Sea urchin	13.4	42.4
Pale kelp	7.4	23.4
Giant kelp stipes	4.8	15.2
**D. Inaccessible vs. Nightingale – Average dissimilarity = 25.3%**		
**Species**	**Average dissimilarity**	**% Contribution**
Giant kelp stipes	8.7	34.5
Pale kelp	6.8	26.8
Sea urchin	4.8	18.8
**E. Inaccessible vs. Tristan – Average dissimilarity = 23.1%**		
**Species**	**Average dissimilarity**	**% Contribution**
Pale kelp	7.0	30.4
Sea urchin	6.2	26.9
Giant kelp stipes	6.2	26.7
**F. Nightingale vs. Tristan – Average dissimilarity = 28.3%**		
**Species**	**Average dissimilarity**	**% Contribution**
Giant kelp stipes	9.6	33.9
Pale kelp	8.9	31.3
Sea urchin	4.0	14.2
