# Supplementary material for: First quantification of subtidal community structure at Tristan da Cunha Islands in the remote South Atlantic: from kelp forests to the deep sea
Source: PLoS One. 2018 Mar 29;13(3):e0195167. doi: 10.1371/journal.pone.0195167 (PMC5875861; doi:10.1371/journal.pone.0195167)
Supplement: S5 Table — Species observed in pelagic BRUVS surveys at Tristan da Cunha group. Total number of individuals recorded using the maximum MaxN per site, and the Mean MaxN per site with associated standard error (SE), and the percentage of sites that species were present. (PDF) [file pone.0195167.s009.pdf]

S5 Table. **Pelagic BRUV taxa and abundance**. Species observed in pelagic BRUVS surveys at Tristan da Cunha group. Total number of individuals recorded using the maximum MaxN per site, and the Mean MaxN per site with associated standard error (SE), and the percentage of sites that species were present.

Common Name	Scientific Names	Maximum MaxN	Mean MaxN	SE	% Sites
**Subantarctic fur seal**	*Arctocephalus tropicalis*	1	0.038	0.038	3.7%
**Loggerhead Turtle**	*Caretta caretta*	1	0.038	0.038	3.7%
**Porbeagle shark**	*Lamna nasus*	1	0.038	0.038	3.7%
**Pilot fish**	*Naucrates ductor*	1	0.038	0.038	3.7%
**Crested bellowfish**	*Notopogon lilliei*	1	0.038	0.038	3.7%
**Blue shark**	*Prionace glauca*	23	0.885	0.199	55.6%
**Oval driftfish**	*Schedophilus velaini*	1	0.038	0.038	3.7%
**Yellowtail amberjack**	*Seriola lalandi*	69	2.654	2.415	7.4%
**Shepherd's beaked whale**	*Tasmacetus shepherdi*	2	0.077	0.075	3.7%
**Striped marlin**	*Kajikia albida*	1	0.038	0.038	3.7%
**Albacore**	*Thunnus alalunga*	2	0.077	0.075	3.7%
**Yellowfin tuna**	*Thunnus albacares*	3	0.115	0.063	11.1%
**Horse mackerel**	*Trachurus* sp	198	7.615	3.687	44.4%
