# Supplementary material for: First quantification of subtidal community structure at Tristan da Cunha Islands in the remote South Atlantic: from kelp forests to the deep sea
Source: PLoS One. 2018 Mar 29;13(3):e0195167. doi: 10.1371/journal.pone.0195167 (PMC5875861; doi:10.1371/journal.pone.0195167)
Supplement: S6 Table — Fish taxa observed on deep-sea drop-cams in the Tristan da Cunha Islands group. Freq.–frequency of occurrence out of all drops (n = 23). (PDF) [file pone.0195167.s010.pdf]

S6 Table. Deep sea fish. Fish taxa observed on deep-sea drop-cams in the Tristan da Cunha Islands group. Freq. – frequency of occurrence out of all drops (*n*=23).

Family	Taxa	Common name	Freq.	Depth range (m)
Berycidae	*Beryx decadactylus*	Roughy/Alfonsino	0.18	656-1203
Carangidae	*Seriola lalandi*	Yellowtail amberjack	0.04	164
Centrolophidae	*Hyperoglyphe antarctica*	Southern Butterfish	0.30	190-708
Centrolophidae	*Schedophilus velaini*	Oval driftfish	0.04	418
Emmelichthyidae	*Emmelichthys nitidus*	Southern rover	0.04	714
Etmopteridae	*Etmopterus* sp. (likely *granulosus*)	Lantern shark	0.56	714-1404
Hexanchidae	*Hexanchus griseus*	Bluntnose sixgill shark	0.30	190-1027
Hexanchidae	*Notorynchus cepedianus*	Sevengill shark	0.04	164
Macrouridae	*Coelorinchus* sp.	Grenadier	0.61	656-1414
Moridae	*Physiculus karrerae*	Deepwater cod	0.39	467-1414
Moridae	*Antimora sp.*	Antimora cod	0.17	994-1414
Ophidiidae	Unidentified	Cusk-eel	0.17	1027-1414
Oreosomatidae	*Neocyttus* sp.	Oreo dory	0.22	994-1200
Phocidae	*Mirounga leonina*	Southern elephant seal	0.04	190
Polyprionidae	*Polyprion oxygeneios*	Wreckfish	0.09	164-418
Sebastidae	*Helicolenus mouchezi*	Soldier	0.22	190-608
Sebastidae	*Sebastes capensis*	False jacopever	0.04	190
Serranidae	*Lepidoperca coatsii*	Seabass	0.04	190
Somniosidae	*Somniosus antarcticus*	Southern sleeper shark	0.04	1027
Synaphobranchidae	*Synaphobranchus* sp. (likely *brevidorsalis*)	Cutthroat eel	0.39	656-1414
Unidentified	Unidentified	Eel sp. 1	0.17	953-1414
